# Supplementary material for: Feasibility and acceptability of offering breast cancer risk assessment to general population women aged 30–39 years: a mixed-methods study protocol
Source: BMJ Open. 2024 Jan 10;14(1):e078555. doi: 10.1136/bmjopen-2023-078555 (PMC10806663; doi:10.1136/bmjopen-2023-078555)
Supplement: Supplementary data [file bmjopen-2023-078555supp001.pdf]

Supplementary file 1. BCAN-RAY risk feedback letters (average, increased)

INSERT LOGOS

Nightingale Centre, Wythenshawe Hospital
Manchester University NHS Foundation Trust
Southmoor Road
Manchester
M23 9LT
Tel: INSERT NUMBER

INSERT PARTICIPANT NAME
INSERT ADDRESS
INSERT ADDRESS
INSERT ADDRESS

INSERT POSTCODE

Date: **INSERT DATE**

Dear **[INSERT NAME]**,

RE: BCAN-RAY Study
NHS number: INSERT

Thank you for taking part in the BCAN-RAY study. This is your first risk feedback letter. A second letter will follow when the study is complete for all women (probably in 2025). It is possible that the second letter may change your risk level.

We have calculated your risk of developing breast cancer in the next 10 years from the following information collected in this study:

- Breast cancer risk factors as assessed from the information you provided on the risk factor questionnaire
- Breast density (the amount of tissue in your breast that is not fat) as assessed from your mammogram
- DNA as assessed from your saliva (spit) sample

Your risk of developing breast cancer in the next 10 years was calculated to be:

Average for the population – that is less than 3 in 100 chance of developing breast cancer in the next 10 years.

More detailed information about your risk result is given in the enclosed document. This information is also available on the study web-based application, which can be accessed by scanning this QR code:

INSERT QR CODE FOR WEB BASED APPLICATION

We also confirm that no pathological variants (mutations) were identified in the 9 risk genes analysed in your saliva sample DNA.

Further information and support resources

There are things that all women can do to reduce their risk of breast cancer, such as maintaining a healthy weight through diet and exercise and limiting alcohol intake. More information on the ways to reduce your risk is provided in the accompanying leaflet. It is also important to regularly check your breasts and report anything new or unusual to a GP. A guide explaining how to check your breasts is enclosed.

Additionally, you may find the following sources of information and support useful if you have any breast health concerns.

CoppaFeel!

Website: https://coppafeel.org/

Breast Cancer Now

Website: https://breastcancernow.org/

They have a section where you can ask any questions you have relating to breast health:

https://forum.breastcancernow.org/t5/Ask-Our-Nurses/ct-p/Asknurses

They also offer a free, confidential helpline to answer questions about breast cancer or breast health – 0808 800 6000 (Text relay prefix – 18001)

Should you have any questions about the study please get in touch with the study team on INSERT NUMBER.

Yours sincerely,

INSERT SIGNATURE
INSERT NAME

INSERT LOGOS

Nightingale Centre, Wythenshawe Hospital
Manchester University NHS Foundation Trust
Southmoor Road
Manchester
M23 9LT
Tel: INSERT NUMBER

INSERT PARTICIPANT NAME
INSERT ADDRESS
INSERT ADDRESS
INSERT ADDRESS

INSERT POSTCODE

Date: **INSERT DATE**

Dear **[INSERT NAME]**,

RE: BCAN-RAY Study
NHS number: INSERT

Thank you for taking part in the BCAN-RAY study. This is your first risk feedback letter.

A second letter will follow when the study is complete for all women (probably in 2025). It is possible that the second letter may change your risk level.

Your result:

You are at **increased** risk of breast cancer

This means that you are more likely to develop breast cancer than other women your age in the general population.

The details of your 10 year risk and lifetime risk of breast cancer compared to the general population are provided in the attached document and are also available on the study web-based application, which can be accessed by scanning this QR code:

INSERT QR CODE FOR WEB BASED APPLICATION

The factors that may have increased your personal risk were:

- Breast cancer risk factors as assessed from the information you provided on the risk factor questionnaire
- Breast density (the amount of tissue in your breast that is not fat) as assessed from your mammogram
- DNA as assessed from your saliva (spit) sample

At this level of risk you will be eligible to start breast screening earlier than the general population and will have access to breast cancer risk reducing approaches.

Gene mutation search

We did not identify a pathological variant (mutation) in any of the 9 risk genes tested.

OR

We have also identified a pathological variant (mutation) in one of the 9 risk genes tested. We would like to give you the opportunity to discuss the potential implications of this for yourself and your family in more detail and the planned risk review appointment (see below) will be with a geneticist (a doctor who specialises in gene mutations and what they mean for families).

Risk review appointment

We would like to offer you a face-to-face appointment at the Family History Risk and Prevention Clinic at The Nightingale Centre to discuss your risk result further. During this appointment, your breast cancer risk will be explained to you along with information about additional breast screening and when this can begin in addition to ways to reduce your risk.

This appointment is part of NHS care and not part of the study itself. As such, a referral into the clinic will be made by your GP and an appointment will be arranged. This should be within 8-12 weeks so if you have not received an appointment 8 weeks after receiving your risk result, please contact the Nightingale team on **INSERT NUMBER**.

Further information and support resources

There are things that all women can do to reduce their risk of breast cancer, such as maintaining a healthy weight through diet and exercise and limiting alcohol intake. More information on the ways to reduce your risk is provided in the accompanying leaflet. It is also important to regularly check your breasts and report anything new or unusual to a GP. A guide explaining how to check your breasts is enclosed.

Additionally, you may find the following sources of information and support useful if you have any breast health concerns.

CoppaFeel!

Website: https://coppafeel.org/

Breast Cancer Now

Website: https://breastcancernow.org/

They have a section where you can ask any questions you have relating to breast health:

https://forum.breastcancernow.org/t5/Ask-Our-Nurses/ct-p/Asknurses

They also offer a free, confidential helpline to answer questions about breast cancer or breast health – 0808 800 6000 (Text relay prefix – 18001)

Should you have any questions about the study please get in touch with the study team on INSERT NUMBER.

Yours sincerely,

INSERT SIGNATURE
INSERT NAME
