# Supplementary material for: Feasibility and acceptability of offering breast cancer risk assessment to general population women aged 30–39 years: a mixed-methods study protocol
Source: BMJ Open. 2024 Jan 10;14(1):e078555. doi: 10.1136/bmjopen-2023-078555 (PMC10806663; doi:10.1136/bmjopen-2023-078555)
Supplement: Supplementary data [file bmjopen-2023-078555supp002.pdf]

Supplementary file 2. Detailed description of self-reported measures of potential harms and benefits of participation in breast cancer risk assessment

Measures	Description
State anxiety (36) and cancer worry (37)	To determine whether increased distress is a harm of participating in breast cancer risk assessment, we will compare levels of general anxiety and breast cancer worry between average and increased risk women and across time to evaluate short as well as longer term effects. One might expect changes in distress, particularly amongst women being identified as increased risk, as the result may be unexpected because of a lack of family history of the disease. General state anxiety will be assessed using the six-item short-form of the state scale of the State Trait Anxiety Inventory (STAI) (36), with participants responding to six statements (e.g. “I feel tense”) about how they currently feel by selecting one of the following response options “not at all”, “somewhat”, “moderately” and “very much”.<br><br>Breast cancer worry will be assessed using the Lerman Cancer Worry Scale (37). The scale consists of six statements such as: “how often do you worry about developing breast cancer?”. Participants will endorse one of the following response options for items 1-3 and 5: “never”, “rarely”, “sometimes”, and “almost all the time”. For items 4 and 6, participants select one option from “not at all”, “a little”, “somewhat”, and “a lot”.

	Both scales have previously been used in similar studies evaluating the psychological impact of receiving breast cancer risk estimates (26, 27).
Risk perception (38)	Perceived comparative risk of developing breast cancer will be assessed using a single item whereby women will be asked to rate their risk of developing breast cancer in the next 10 years, compared with other women of their age (38). Participants will select one of the following response options: “much higher”, “a bit higher”, “about the same”, “a bit lower”, and “much lower”.
Attitudes towards breast cancer risk assessment (39)	Attitudes towards breast cancer risk assessment will be assessed following a standard approach (39). Three items will be used to assess affective (feelings towards the behaviour) and instrumental (evaluation of the behaviour’s outcomes) attitudes. Women will be asked to indicate the extent to which they view risk assessment as good/beneficial/important, with response options including: “entirely good”, “mainly good”, “neither good nor bad”, “mainly bad”, and “entirely bad”.
Knowledge	No validated measure has been developed for the assessment of breast cancer risk assessment knowledge. Therefore, we decided to create a measure focusing on knowledge of the breast cancer risk assessment process to assess the potential benefit of increased knowledge and inform future implementation. The measure is informed by data on potential misunderstandings of the breast cancer risk assessment process

	identified from a content analysis of qualitative data collected in the context of optimising the delivery of breast cancer risk assessment in the BCAN-RAY study (28). The measure consists of three questions that map onto the potential misunderstandings identified, namely eligibility for risk assessment, the purpose of the mammogram and access to screening and preventive strategies. Subjective knowledge will be assessed with a single item that asks women to rate how informed they feel about their breast cancer risk, from "very well informed", "quite well informed", "quite uninformed", and "not very informed at all".
Satisfaction with risk feedback information (40)	Satisfaction with risk feedback information will be assessed using four items from a published scale (40) that has been used previously in breast cancer risk-stratification research (26, 27). Women will be asked how well informed they feel about their breast cancer risk, how satisfied they are with the amount of information given, how confusing they found it, and how clear they found the information. Participants will select one of the following response options for each item: "strongly agree", "agree", "agree somewhat", "undecided", "somewhat disagree", "disagree", and "strongly disagree.
Satisfaction with decision to participate in breast cancer risk assessment (41)	Participants' remorse or distress over their decision to take part in breast cancer risk assessment will be assessed using a single item adapted from the Decision Regret Scale (41): "The decision to participate in

	breast cancer risk assessment was a good decision for me". Response options will be "strongly agree", "agree", "neither agree nor disagree", "disagree", and "strongly disagree".
