# Supplementary material for: Feasibility and acceptability of offering breast cancer risk assessment to general population women aged 30–39 years: a mixed-methods study protocol
Source: BMJ Open. 2024 Jan 10;14(1):e078555. doi: 10.1136/bmjopen-2023-078555 (PMC10806663; doi:10.1136/bmjopen-2023-078555)
Supplement: Supplementary data [file bmjopen-2023-078555supp003.pdf]

Supplementary file 3. Participant questionnaires (baseline, 6 weeks post risk feedback and 6 months post risk feedback)

Breast **CAN**cer **– R**isk Assessment in **Y**oung Women **(BCAN-RAY):** Acceptability survey (baseline)

Please enter your unique identifier and date of birth. Your unique identifier can be found on your study invite letter.

Unique study identifier:

Date of birth:

SECTION A – YOUR MENTAL WELL-BEING

A number of statements which people have used to describe how they feel are given below. Please read each of the 6 statements and then circle the most appropriate number below the statement to indicate how you feel right now, at this moment. There are no right or wrong answers. Do not spend too much time on any one statement but give the answer which seems to describe your present feelings best.

A1 **I feel calm**

Not at all	Somewhat	Moderately	Very much
1	2	3	4

A2 **I am tense**

Not at all	Somewhat	Moderately	Very much
1	2	3	4

A3 **I feel upset**

Not at all	Somewhat	Moderately	Very much
1	2	3	4

A4 **I am relaxed**

Not at all	Somewhat	Moderately	Very much
1	2	3	4

A5 **I feel content**

Not at all	Somewhat	Moderately	Very much
1	2	3	4

A6 **I am worried**

Not at all	Somewhat	Moderately	Very much
1	2	3	4

SECTION B – YOUR WORRIES ABOUT DEVELOPING BREAST CANCER
Please read the statements below and circle the number below each statement that best indicates your current level of worry about getting breast cancer someday:

B1 **How often have you thought about your chances of getting breast cancer?**

Never	Rarely	Sometimes	Almost all the time
1	2	3	4

B2 **How often have these thoughts affected your mood?**

Never	Rarely	Sometimes	Almost all the time
1	2	3	4

B3 **How often have these thoughts interfered with your ability to do daily activities?**

Never	Rarely	Sometimes	Almost all the time
1	2	3	4

B4 **How concerned are you about the possibility of getting breast cancer one day?**

Not at all	A little	Somewhat	A lot
1	2	3	4

B5 **How often do you worry about developing breast cancer?**

Never	Rarely	Sometimes	Almost all the time
1	2	3	4

B6 **How much of a problem is this worry?**

Not at all	A little	Somewhat	A lot
1	2	3	4

SECTION C – YOUR PERCEPTION OF BREAST CANCER RISK
Please tick **ONE** of the statements below that best describes your breast cancer risk in relation to other women of a similar age:

C1 **Compared to other women my age, I believe my risk of developing breast cancer in the next 10 years is…**

- ❑ Much higher
- ❑ A bit higher
- ❑ About the same
- ❑ A bit lower
- ❑ Much lower

SECTION D – YOUR ATTITUDES TOWARD BREAST CANCER RISK ASSESSMENT
Please read the statement and items below and circle the number that best indicates how you feel about participating in breast cancer risk assessment right now, at this moment:

D1 **Taking part in breast cancer risk assessment will be…**

Entirely good	Mainly good	Neither good nor bad	Mainly bad	Entirely bad
1	2	3	4	5
Entirely beneficial	Mainly beneficial	Neither beneficial nor harmful	Mainly harmful	Entirely harmful
1	2	3	4	5
Entirely important	Mainly important	Neither important nor unimportant	Mainly unimportant	Entirely unimportant
1	2	3	4	5

SECTION E – INTEREST IN INTERVIEW

We would like to hear more about your experience of participating in breast cancer risk assessment as part of the BCAN-RAY study. Please tick one box to indicate whether you are happy to be contacted about participating in an interview (over the phone or face-to-face).

E1 **I am happy to be contacted about participating in an interview following receipt of my risk results**

YES ❑ NO ❑

Thank you for completing this questionnaire.

Please return your completed questionnaire to the study team in the pre-paid envelope provided.

Sources of information and support

You may find some of the following sources of information and support useful if you have any concerns about breast health.

CoppaFeel!

Website: https://coppafeel.org/

They have a section that provides guidance on checking your breasts: https://self-checkout.coppafeel.org/onboarding

Breast Cancer Now

Website: https://breastcancernow.org/

They have a section where you can ask any questions you have relating to breast health:

https://forum.breastcancernow.org/t5/Ask-Our-Nurses/ct-p/Asknurses

They also offer a free, confidential helpline to answer questions about breast cancer or breast health – 0808 800 6000 (Text relay prefix – 18001)

Breast **CAN**cer **–** **R**isk Assessment in **Y**oung Women **(BCAN-RAY):**
Acceptability survey (6 weeks post risk feedback)

Please enter your unique identifier and date of birth. Your unique identifier can be found on your study invite letter.

Unique study identifier:

Date of birth:

SECTION A – YOUR MENTAL WELL-BEING

A number of statements which people have used to describe how they feel are given below. Please read each of the 6 statements and then circle the most appropriate number below the statement to indicate how you feel right now, at this moment. There are no right or wrong answers. Do not spend too much time on any one statement but give the answer which seems to describe your present feelings best.

A1 **I feel calm**

Not at all	Somewhat	Moderately	Very much
1	2	3	4

A2 **I am tense**

Not at all	Somewhat	Moderately	Very much
1	2	3	4

A3 **I feel upset**

Not at all	Somewhat	Moderately	Very much
1	2	3	4

A4 **I am relaxed**

Not at all	Somewhat	Moderately	Very much
1	2	3	4

A5 **I feel content**

Not at all	Somewhat	Moderately	Very much
1	2	3	4

A6 **I am worried**

Not at all	Somewhat	Moderately	Very much
1	2	3	4

SECTION B – YOUR WORRIES ABOUT DEVELOPING BREAST CANCER
Please read the statements below and circle the number below each statement that best indicates your current level of worry about getting breast cancer someday:

B1 **How often have you thought about your chances of getting breast cancer?**

Never	Rarely	Sometimes	Almost all the time
1	2	3	4

B2 **How often have these thoughts affected your mood?**

Never	Rarely	Sometimes	Almost all the time
1	2	3	4

B3 **How often have these thoughts interfered with your ability to do daily activities?**

Never	Rarely	Sometimes	Almost all the time
1	2	3	4

B4 **How concerned are you about the possibility of getting breast cancer one day?**

Not at all	A little	Somewhat	A lot
1	2	3	4

B5 **How often do you worry about developing breast cancer?**

Never	Rarely	Sometimes	Almost all the time
1	2	3	4

B6 **How much of a problem is this worry?**

Not at all	A little	Somewhat	A lot
1	2	3	4

SECTION C – YOUR PERCEPTION OF BREAST CANCER RISK
Please tick **ONE** of the statements below that best describes your breast cancer risk in relation to other women of a similar age:

C1 **Compared to other women my age, I believe my risk of developing breast cancer in the next 10 years is…**

- ❑ Much higher
- ❑ A bit higher
- ❑ About the same
- ❑ A bit lower
- ❑ Much lower

SECTION D – YOUR BREAST CANCER RISK KNOWLEDGE
Please read the statement below and then circle the most appropriate number below the statement to indicate how informed you feel about your breast cancer risk at this moment:

D1 **How informed do you feel about your breast cancer risk?**

Very well informed	Quite well informed	Quite uninformed	Not very informed at all
1	2	3	4

SECTION E – YOUR KNOWLEDGE
For each question please place **ONE** tick in the box that corresponds with your knowledge/understanding of breast cancer risk assessment being offered in the BCAN-RAY study.

E1 **Who are the intended participants of breast cancer risk assessment in the BCAN-RAY study?**

- ❑ Women who have been told by a healthcare professional that they have a strong family history of breast cancer
- ❑ Women who have not been told by a healthcare professional that they have a strong family history of breast cancer

E2 **What is the purpose of the low dose mammogram in the BCAN-RAY study?**

- ❑ To assess breast density (the amount of tissue in your breast that is not fat)
- ❑ To detect breast cancer

E3 **Who will be given the opportunity to discuss additional breast screening and risk reducing measures with a clinician in the BCAN-RAY study?**

- ❑ Only women identified as being at increased risk of breast cancer
- ❑ All women who participate in the study

SECTION F – YOUR PERCEPTIONS OF THE BREAST CANCER INFORMATION ENCLOSED WITH YOUR RISK FEEDBACK

Thinking about the letter and leaflets you received when you were provided with your risk of developing breast cancer in the next 10 years, please read each statement and then circle the most appropriate number below the statement to indicate how you feel about the information *(please circle **only one number**).*

F1 **I feel well informed about my breast cancer risk.**

Strongly agree	Agree	Somewhat agree	Undecided	Disagree somewhat	Disagree	Strongly disagree
1	2	3	4	5	6	7

F2 **I feel satisfied with the amount of information I have been given.**

Strongly agree	Agree	Somewhat agree	Undecided	Disagree somewhat	Disagree	Strongly disagree
1	2	3	4	5	6	7

F3 **I am confused by the information I have been given.**

Strongly agree	Agree	Somewhat agree	Undecided	Disagree somewhat	Disagree	Strongly disagree
1	2	3	4	5	6	7

F4 **The information was clear.**

Strongly agree	Agree	Somewhat agree	Undecided	Disagree somewhat	Disagree	Strongly disagree
1	2	3	4	5	6	7

Thank you for completing this questionnaire.

Please return your completed questionnaire to the study team in the pre-paid envelope provided.

Sources of information and support

You may find some of the following sources of information and support useful if you have any concerns about breast health.

CoppaFeel!

Website: https://coppafeel.org/

They have a section that provides guidance on checking your breasts:
https://self-checkout.coppafeel.org/onboarding

Breast Cancer Now

Website: https://breastcancernow.org/

They have a section where you can ask any questions you have relating to breast health:

https://forum.breastcancernow.org/t5/Ask-Our-Nurses/ct-p/Asknurses

They also offer a free, confidential helpline to answer questions about breast cancer or breast health – 0808 800 6000 (Text relay prefix – 18001)

Breast **CAN**cer **– R**isk Assessment in **Y**oung Women **(BCAN-RAY):** Acceptability survey (6 months post risk feedback)

Please enter your unique identifier and date of birth. Your unique identifier can be found on your study invite letter.

Unique study identifier:

Date of birth:

SECTION A – YOUR MENTAL WELL-BEING

A number of statements which people have used to describe how they feel are given below. Please read each of the 6 statements and then circle the most appropriate number below the statement to indicate how you feel right now, at this moment. There are no right or wrong answers. Do not spend too much time on any one statement but give the answer which seems to describe your present feelings best.

A1 **I feel calm**

Not at all	Somewhat	Moderately	Very much
1	2	3	4

A2 **I am tense**

Not at all	Somewhat	Moderately	Very much
1	2	3	4

A3 **I feel upset**

Not at all	Somewhat	Moderately	Very much
1	2	3	4

A4 **I am relaxed**

Not at all	Somewhat	Moderately	Very much
1	2	3	4

A5 **I feel content**

Not at all	Somewhat	Moderately	Very much
1	2	3	4

A6 **I am worried**

Not at all	Somewhat	Moderately	Very much
1	2	3	4

SECTION B – YOUR WORRIES ABOUT DEVELOPING BREAST CANCER
Please read the statements below and circle the number below each statement that best indicates your current level of worry about getting breast cancer someday:

B1 **How often have you thought about your chances of getting breast cancer?**

Never	Rarely	Sometimes	Almost all the time
1	2	3	4

B2 **How often have these thoughts affected your mood?**

Never	Rarely	Sometimes	Almost all the time
1	2	3	4

B3 **How often have these thoughts interfered with your ability to do daily activities?**

Never	Rarely	Sometimes	Almost all the time
1	2	3	4

B4 **How concerned are you about the possibility of getting breast cancer one day?**

Not at all	A little	Somewhat	A lot
1	2	3	4

B5 **How often do you worry about developing breast cancer?**

Never	Rarely	Sometimes	Almost all the time
1	2	3	4

B6 **How much of a problem is this worry?**

Not at all	A little	Somewhat	A lot
1	2	3	4

SECTION C – YOUR PERCEPTION OF BREAST CANCER RISK
Please tick **ONE** of the statements below that best describes your breast cancer risk in relation to other women of a similar age:

C1 **Compared to other women my age, I believe my risk of developing breast cancer in the next 10 years is…**

- ☐ Much higher
- ☐ A bit higher
- ☐ About the same
- ☐ A bit lower
- ☐ Much lower

SECTION D – YOUR ATTITUDES TOWARD BREAST CANCER RISK ASSESSMENT
Please read the statement and items below and circle the number that best indicates how you feel about participating in breast cancer risk assessment right now, at this moment:

D1 **Taking part in breast cancer risk assessment was…**

Entirely good	Mainly good	Neither good nor bad	Mainly bad	Entirely bad
1	2	3	4	5
Entirely beneficial	Mainly beneficial	Neither beneficial nor harmful	Mainly harmful	Entirely harmful
1	2	3	4	5
Entirely important	Mainly important	Neither important nor unimportant	Mainly unimportant	Entirely unimportant
1	2	3	4	5

SECTION E – YOUR SATISFACTION WITH DECISION TO PARTICIPATE IN BREAST CANCER RISK ASSESSMENT

Please read the statement below and then circle the most appropriate number below the statement to indicate how satisfied you are with your decision to participate in breast cancer risk assessment.

E1 **The decision to participate in breast cancer risk assessment was a good decision for me**

Strongly agree	Agree	Neither agree nor disagree	Disagree	Strongly disagree
1	2	3	4	5

Thank you for completing this questionnaire.

Please return your completed questionnaire to the study team in the pre-paid envelope provided.

Sources of information and support

You may find some of the following sources of information and support useful if you have any concerns about breast health.

CoppaFeel!

Website: https://coppafeel.org/

They have a section that provides guidance on checking your breasts: https://self-checkout.coppafeel.org/onboarding

Breast Cancer Now

Website: https://breastcancernow.org/

They have a section where you can ask any questions you have relating to breast health:

https://forum.breastcancernow.org/t5/Ask-Our-Nurses/ct-p/Asknurses

They also offer a free, confidential helpline to answer questions about breast cancer or breast health – 0808 800 6000 (Text relay prefix – 18001)
