# Supplementary material for: Feasibility and acceptability of offering breast cancer risk assessment to general population women aged 30–39 years: a mixed-methods study protocol
Source: BMJ Open. 2024 Jan 10;14(1):e078555. doi: 10.1136/bmjopen-2023-078555 (PMC10806663; doi:10.1136/bmjopen-2023-078555)
Supplement: Supplementary data [file bmjopen-2023-078555supp004.pdf]

**Supplementary file 4.** Interview topic guide

**Opening questions**

As you know, we are interested in what women think about the offer of finding out their breast cancer risk as part of the BCAN-RAY study. To start, can you tell me anything about whether breast cancer risk is something you have thought about before being invited to join the BCAN-RAY study?

I understand you were invited to have your breast cancer risk assessed; can we go back to that point and tell me what that was like? What did you think at that point?

How did you make the decision to take part in breast cancer risk assessment?
Prompts:

- Were there are aspects of the BCAN-RAY study that made you question whether to take part (any concerns)?
- Can you tell me anything about why you wanted to know your risk? Anything personal to you?
- How did you receive the invite (as a letter from GP practice if no recall)? What do you think about receiving it that way? How do you think that influenced your decision to have your breast cancer risk assessed?
- (if not already come up) When you were deciding, did you discuss it with anyone (friend / family / study team / GP)?
- Did you feel you had all the information you needed to make a decision about whether to take part? If not, what would have been helpful to know?

**Questions relating to risk assessment process**

Can you tell me what you had to do once you joined the study? Could you tell me about what happened when you had your breast cancer risk assessed?
Probes: What was it like / can you tell me anything about it
Prompts:

- Completing the risk factors questionnaire e.g. how easy was it to access, can you remember what it was asking you to do, were any questions unclear, ability to answer the questions more generally, did you find any questions uncomfortable to answer, did you get any support to help with this part of the study
- What happened once you completed the questionnaire? What was that time-period like?
- Attending the appointment at the hospital (spit sample, mammogram) e.g. what did you think about how the appointment was arranged
- Waiting for the risk feedback results (up to 16 weeks turnaround) e.g. how were you feeling during this time, what did you think about the length of time you had to wait, did you look for any information related to breast cancer during this time
- Receiving the risk feedback (a letter if no recall)
- Contents/wording of the letter (thoughts, feelings and understanding) e.g. did the feedback you received match your expectations in terms of what you thought you would be told
- Logging back into the app to view detailed risk feedback (if not, why not)

- Personal meaning of risk category received e.g. what do you remember about your risk result, how would you describe the risk, how do you feel about the factors that contributed to your risk (increased risk), how did it make you feel, was it something you expected, how do you feel about your risk today/now
- Discussing risk feedback with others (friends / family / healthcare professionals)
  - Did you talk about your risk feedback with anyone in the study team / outside the study team? If yes/no, why? What did you discuss?
  - What did you think of the support provided at this point?
- (increased risk) Experience of risk consultation
  - What did you think about the option to receive an appointment to discuss your risk if it was increased?

After you received your risk feedback, did you do anything differently that you thought might reduce your breast cancer risk?
Prompts:

- (all) Health behaviours
- (increased risk) Recommendation to contact medical doctors to discuss risk reducing medication / additional screening
- (increased risk) Deciding whether to have risk reducing medication
- (increased risk) Deciding whether to have additional screening

Looking back, was there anything that caused any concerns during the risk assessment process? Is there anything you would have preferred to happen in a different way?

Looking back, how do you feel about having made the decision to take part in breast cancer risk assessment?
Prompts:

- Did you understand what was involved when you made the decision to participate?
  Probe: did you have sufficient information?

The way breast cancer risk is calculated changes over time as we learn more about new risk factors. As we are trying to find out whether using a low dose mammogram helps to identify younger women at risk of developing breast cancer, towards the end of the study you will receive updated risk feedback. At this point, your risk might change. What are your thoughts about this? Why?

We are trying to figure out whether introducing a breast cancer risk assessment service for women aged 30 to 39 years is a good or bad idea. What are your thoughts about this? Why? Would you recommend a breast cancer risk assessment service to friends and family members of a similar age?

**Finishing comments**

Thanks for your time today. We do really appreciate it.

- Is there anything else you want to add?

- Is there anything you thought you would talk about today which you haven't had a chance to say and want to mention?
- Do you have any questions for me?

Thanks again. The interview will be typed up by a partner transcription company we use. When this is done, we will remove anything you have said that could identify you such as names or places and you will be given a fake name. If you have any questions feel free to contact the research team at any time *[point out contact details]*.
