# Supplementary material for: Feasibility and acceptability of offering breast cancer risk assessment to general population women aged 30–39 years: a mixed-methods study protocol
Source: BMJ Open. 2024 Jan 10;14(1):e078555. doi: 10.1136/bmjopen-2023-078555 (PMC10806663; doi:10.1136/bmjopen-2023-078555)
Supplement: Author's manuscript [file bmjopen-2023-078555.draft_revisions.pdf]

BMJ Open is committed to open peer review. As part of this commitment we make the peer review history of every article we publish publicly available.

When an article is published we post the peer reviewers' comments and the authors' responses online. We also post the versions of the paper that were used during peer review. These are the versions that the peer review comments apply to.

The versions of the paper that follow are the versions that were submitted during the peer review process. They are not the versions of record or the final published versions. They should not be cited or distributed as the published version of this manuscript.

BMJ Open is an open access journal and the full, final, typeset and author-corrected version of record of the manuscript is available on our site with no access controls, subscription charges or pay-per-view fees (http://bmjopen.bmj.com).

If you have any questions on BMJ Open's open peer review process please email info.bmjopen@bmj.com

BMJ Open

# The feasibility and acceptability of offering breast cancer risk assessment to general population women aged 30-39 years: A mixed-methods study protocol

| | |
|---|---|
| Journal: | *BMJ Open* |
| Manuscript ID | bmjopen-2023-078555 |
| Article Type: | Protocol |
| Date Submitted by the Author: | 04-Aug-2023 |
| Complete List of Authors: | Hindmarch, Sarah; The University of Manchester, Manchester Centre for Health Psychology, Division of Psychology and Mental Health, School of Health Sciences, Faculty of Biology, Medicine and Health<br>Howell, Sacha; The University of Manchester, Division of Cancer Sciences, Faculty of Biology, Medicine and Health, University of Manchester, Manchester Academic Health Science Centre<br>Usher-Smith, Juliet; University of Cambridge, Primary Care Unit, Department of Public Health and Primary Care<br>Gorman, Louise; The University of Manchester, NIHR Greater Manchester Patient Safety Research Collaboration, Division of Population Health, Health Services Research & Primary Care, Faculty of Biology, Medicine and Health<br>Evans, D. Gareth ; The University of Manchester, Manchester Academic Health Science Centre, Division of Evolution and Genomic Sciences, School of Biological Sciences, Faculty of Biology, Medicine and Health<br>French, David; The University of Manchester, Manchester Centre for Health Psychology, Division of Psychology and Mental Health, School of Health Sciences, Faculty of Biology, Medicine and Health |
| Keywords: | Breast tumours < ONCOLOGY, Feasibility Studies, QUALITATIVE RESEARCH, Risk Factors, Surveys and Questionnaires, Health Equity |
| | |

SCHOLARONE™
Manuscripts

# The feasibility and acceptability of offering breast cancer risk assessment to general population women aged 30-39 years: A mixed-methods study protocol

Sarah Hindmarch[1*]; Sacha J. Howell[2]; Juliet A. Usher-Smith[3]; Louise Gorman[4]; D. Gareth Evans[5,6]; David P. French[1]

[1]Manchester Centre for Health Psychology, Division of Psychology and Mental Health, School of Health Sciences, Faculty of Biology, Medicine and Health, University of Manchester, Manchester, UK

[2]Division of Cancer Sciences, Faculty of Biology, Medicine and Health, University of Manchester, Manchester Academic Health Science Centre, Manchester, UK

[3]Primary Care Unit, Department of Public Health and Primary Care, University of Cambridge, Cambridge, UK

[4]NIHR Greater Manchester Patient Safety Research Collaboration, Division of Population Health, Health Services Research & Primary Care, Faculty of Biology, Medicine and Health, University of Manchester, Manchester, UK

[5]University of Manchester, Manchester Academic Health Science Centre, Division of Evolution and Genomic Sciences, School of Biological Sciences, Faculty of Biology, Medicine and Health, Manchester, UK

[6]St Mary's Hospital, Manchester University NHS Foundation Trust, Manchester Academic Health Science Centre, North West Genomics Laboratory Hub, Manchester Centre for Genomic Medicine, Manchester, UK

Corresponding author, sarah.hindmarch@postgrad.manchester.ac.uk



For peer review only - http://bmjopen.bmj.com/site/about/guidelines.xhtml

Word count: 4,458

## Abstract

**Introduction:** Breast cancer incidence starts to increase exponentially when women reach 30-39 years, hence before they are eligible for breast cancer screening. The introduction of breast cancer risk assessment for this age group could lead to those at higher risk receiving benefits of earlier screening and preventive strategies. Currently, risk assessment is limited to women with family history of breast cancer only. The BCAN-RAY study is evaluating a comprehensive breast cancer risk assessment strategy for women aged 30-39 years incorporating a questionnaire of breast cancer risk factors, low-dose mammography to assess breast density, and polygenic risk. The present study will assess the feasibility and acceptability of the BCAN-RAY risk assessment strategy.

**Methods and analysis:** The present study involves women undergoing risk assessment as part of the BCAN-RAY case-control study (*n* = 750). They will be aged 30-39 years without a strong family history of breast cancer and invited to participate via general practice. A comparison of uptake rates by socioeconomic status and ethnicity between women who participate in the BCAN-RAY study and women who decline participation will be conducted. All participants will be asked to complete self-report questionnaires to assess key potential harms including increased state anxiety (STAI), cancer worry (Lerman Cancer Worry Scale), and satisfaction with decision to participate (Decision Regret Scale), alongside potential benefits such as feeling more informed about breast cancer risk. A sub-sample of approximately 24 women (12 at average risk and 12 at increased risk) will additionally participate in semi-structured interviews to understand the acceptability of the risk assessment strategy and identify any changes needed to it to increase uptake.



For peer review only - http://bmjopen.bmj.com/site/about/guidelines.xhtml

**Ethics and dissemination:** Ethical approval was granted by North West - Greater Manchester West Research Ethics Committee (reference: 22/NW/0268). Study results will be disseminated through peer-reviewed journals, conference presentations and charitable organisations.

**Trial registration:** NCT05305963.

**Keywords:** risk assessment, breast cancer, psychological impact, health inequalities, acceptability

For peer

## Article Summary

**Strengths and limitations of this study**

- This is the first study to examine the feasibility and acceptability of comprehensive breast cancer risk assessment for general population women aged 30-39 years.
- This study uses a mixed methods design; the combination of qualitative and quantitative data will result in a more comprehensive understanding of the processes affecting implementation.
- The findings of this study will identify modifications needed to the breast cancer risk assessment strategy to increase the likelihood of future implementation studies being successful.
- Outcome measures assessing potential harms and benefits of participating in breast cancer risk assessment will be collected at three timepoints, allowing for assessment of short and long term effects.



For peer review only - http://bmjopen.bmj.com/site/about/guidelines.xhtml

- The quality and completeness of ethnicity data across general practices may be suboptimal for the planned analyses.

## Introduction

Breast cancer is the most common cancer diagnosed worldwide for women, with increasing incidence rates observed in pre-menopausal women in recent years (1, 2). This is concerning as breast cancer is more frequently lethal in younger women than in those diagnosed aged over 50 years (10-year survival aged <40 years at diagnosis 70% vs 87% in those >50 years) (3). This is due to a combination of factors, notably later stage at presentation and a greater proportion of women developing more aggressive breast cancer subtypes (4-6). Breast cancer is the leading cause of death in women aged 35-50 years in the UK (7). Therefore, there is a pressing need to identify younger women at increased risk of developing breast cancer so they can be offered screening and preventive strategies (8).

Assessment of an individual's breast cancer risk is one proposed approach for identifying young women eligible for screening and preventive strategies (9). In the UK, a strong family history of breast cancer or known high risk genetic variant in a close relative is the only criteria by which women aged under 50 years can access screening and preventive strategies prior to a diagnosis of breast cancer (10). However, at least 65% of women who develop breast cancer before the age of 50 years do not have such a family history and are not currently identified as being at increased risk (3, 11).



For peer review only - http://bmjopen.bmj.com/site/about/guidelines.xhtml

The reliance on family history belies the progress over recent decades in the identification of additional breast cancer risk factors including those related to reproductive and hormonal history, alcohol consumption, polygenic risk scores and mammographic density. These additional factors have been incorporated into risk prediction models, resulting in improved discrimination across all age groups (12-15). In the UK, the PROCAS study confirmed it was possible to accurately estimate a woman's individual risk of developing breast cancer at the time of mammographic screening using a self-reported questionnaire of breast cancer risk factors and assessment of mammographic density and polygenic risk (16). Using this comprehensive approach to risk assessment identified 18% of women as being at least moderate risk of developing breast cancer in comparison to only 3.7% using family history alone (17). Therefore, a greater number of women were identified who would be eligible for consideration of screening and preventive strategies (10). Trials are underway internationally to establish the potential effectiveness of risk-based screening strategies for women attending breast cancer screening programmes over the age of 40 years (18, 19). However, inclusion of breast cancer risk assessment at the time of national mammographic screening programmes will miss younger women eligible for screening and preventive strategies. Therefore, the introduction of comprehensive breast cancer risk assessment from an earlier age is currently being considered.

A recent review determined that breast cancer risk assessment for women under 50 years currently satisfies many of the standard principles for screening (20). However, uncertainties remain with respect to the optimal strategy for implementation and potential impact of the invitation process on health inequalities. The Breast CANcer Risk Assessment in Younger Women (BCAN-RAY) case-control study (NCT05305963) aims to evaluate a



For peer review only - http://bmjopen.bmj.com/site/about/guidelines.xhtml

comprehensive breast cancer risk assessment strategy amongst a diverse ethnic and socioeconomic population of women aged 30-39 years without a strong family history of breast cancer (21). The BCAN-RAY study aims to primarily assess the impact of mammographic density on breast cancer risk in this age group. To address this, we have developed a low-dose mammogram technique which uses a tenth or less of the radiation dose of a full dose screening mammogram making it safer. Furthermore, an automated method of analysis not requiring radiologist review will be utilised, removing the risk of unnecessary recall for additional imaging. This approach has been shown to be accurate in younger women (22).

The risk assessment strategy thereby consists of a questionnaire of breast cancer risk factors, low-dose mammography to measure mammographic density, and a saliva sample to assess polygenic risk and the presence of pathogenic variants in high and moderate-risk genes. The breast cancer risk assessment strategy adopted in the BCAN-RAY study is herein referred to as the BCAN-RAY approach. Women with a strong family history of breast cancer are ineligible to participate because they can access screening and preventive strategies through referral to Family History, Risk and Prevention Clinics (FHRPCs). Women identified as being at increased risk will be offered an appointment at a FHRPC to discuss their risk result further and potential management options. Options in the UK include access to breast screening from the age of 40 years (if 10-year risk reaches 3% by 40) and preventive strategies such as weight loss or weight gain prevention interventions and risk-reducing medication. Uptake of these screening and preventive strategies by younger women has the potential to facilitate earlier detection of breast cancer and reduce breast cancer mortality (9).



For peer review only - http://bmjopen.bmj.com/site/about/guidelines.xhtml

In line with the MRC Framework for Developing and Evaluating Complex Interventions (23), it is imperative to assess the feasibility of the BCAN-RAY approach in order to inform future decisions about implementation. One key consideration is a need to assess whether the invitation process exacerbates health inequalities through lower recruitment of ethnic minority populations and women from low socioeconomic backgrounds. Previous efforts to implement risk assessment at the time of mammographic screening have demonstrated these problems (24). This is important to consider as addressing ethnic disparities in breast cancer mortality has been recognised as a key research priority (25).

Secondly, potential harms and benefits need to be identified. There is now considerable evidence on the effects of providing breast cancer risk estimates to women aged 47-73 years recruited via the NHS Breast Screening Programme. These data indicate that women subsequently had more accurate perceptions of risk with no evidence of significant adverse effects on anxiety or cancer worry (26, 27). Nevertheless, there is a need to show an absence of adverse effects when setting up a new programme with younger women for several reasons. First, one might expect more acute distress amongst younger women at increased risk as the result may be more unexpected because of a lack of family history of the disease, suggesting anxiety and cancer worry are important outcomes to assess. Second, due to the potential implications of being identified as at increased risk for younger women in terms of reproductive decision-making, a possible harm could be that participants experience remorse or distress over their decision to take part in breast cancer risk assessment. In terms of benefits, it is anticipated that women will feel more informed about



For peer review only - http://bmjopen.bmj.com/site/about/guidelines.xhtml

breast cancer risk as a result of participation which will enable them to make informed choices about subsequent risk management options.

Finally, it is important to consider acceptability of the BCAN-RAY approach to women aged 30-39 years to optimise the likelihood of future implementation being successful. If the processes of identification, risk assessment and feedback are unacceptable, then the potential benefits will not be realised. We have previously conducted a qualitative study with women aged 30-39 years which suggested that undergoing breast cancer risk assessment was acceptable in principle (28). However, risk assessment was presented as a hypothetical prospect in that study so how women may respond once they have experienced it and any changes required to increase engagement and uptake remain unknown.

The present study aims to examine the feasibility and acceptability of a strategy to offer breast cancer risk assessment to women aged 30-39 years in a diverse ethnic and socioeconomic geographical region. A mixed-methods approach will be adopted in order to capitalise on the strengths of both quantitative and qualitative methods, resulting in a more comprehensive understanding of the processes affecting implementation (29). Specific objectives of this study are to:

a) Examine uptake rates according to socioeconomic status and ethnicity to determine impact of the invitation process on health inequalities
b) Identify potential harms and benefits of participation in breast cancer risk assessment
c) Understand the acceptability of the BCAN-RAY approach



For peer review only - http://bmjopen.bmj.com/site/about/guidelines.xhtml

# Methods

## Design

BCAN-RAY is a case-control study (21). Approximately one thousand women will be recruited between May 2023 and May 2025, 250 women diagnosed with breast cancer when they were aged 30-39 years (cases) and 750 controls currently aged 30-39 years without a strong family history of breast cancer. The present feasibility study involves the control participants only and uses three different designs to address the three objectives.

### *a. Health inequalities assessment*

A between-subjects comparison will be made between women who participate in the BCAN-RAY study and women who decline participation according to socioeconomic status and ethnicity.

### *b. Identification of potential harms and benefits*

Quantitative questionnaires will be administered to each woman at three timepoints; baseline, 6 weeks post risk feedback and 6 months post risk feedback. A between-subjects comparison will be made between average and increased risk women for outcomes assessed at multiple timepoints.

### *c. Understanding acceptability*

A cross-sectional qualitative design will be adopted employing one-to-one semi-structured interviews.

## Setting and participants



For peer review only - http://bmjopen.bmj.com/site/about/guidelines.xhtml

All general practices across Greater Manchester have been approached for participation in BCAN-RAY as participant identification centres. An electronic database search will be conducted by each practice to identify women aged 30-39 years predicted to meet eligibility criteria. All potentially eligible women will be invited. We expect to recruit a diverse sample in terms of ethnicity and socioeconomic status given that Greater Manchester has one of the most ethnically diverse populations in the UK in addition to some of the most deprived areas (30, 31). Furthermore, general practices in areas of higher ethnic and socioeconomic diversity will be prioritised during setup. Participants meet BCAN-RAY study inclusion criteria if they are (1) born biologically female, (2) aged 30-39 years, and (3) able to provide informed consent. Participants cannot take part if they meet any of the exclusion criteria outlined in Table 1.

**Table 1.** Study exclusion criteria

| |
|---|
| Strong family history of breast cancer, defined as a first degree relative diagnosed with breast cancer under the age of 50 or two or more second-degree relatives diagnosed with breast cancer at any age |
| Already under follow up in a breast cancer family history clinic or have a known mutation in a moderate or high-risk breast cancer gene |
| Any prior malignancy (excluding non-melanoma skin cancer) |
| Had a double mastectomy (both breasts removed) |
| Breast implants or breast augmentation surgery |
| Currently pregnant |
| Currently breast-feeding or stopped breast-feeding less than six months ago |



For peer review only - http://bmjopen.bmj.com/site/about/guidelines.xhtml

Any condition that would make breast cancer risk assessment inappropriate such as a severe psychiatric or physical illness (assessed by the individual responsible for identifying and inviting women)

Unable to understand written English

---

## Procedure

### ***BCAN-RAY study***

Participating general practices will send postal invitations to eligible women. The BCAN-RAY invitation letter will contain a QR code and web-link to access the participant information sheet and instructions directing prospective participants to the risk assessment web-based application. Once participants have consented to the study online, they will be directed to the BCAN-RAY risk factors questionnaire based on the Tyrer-Cuzick algorithm (32). Participants will be able to answer part of the questionnaire, save and return to it at a later date. If a participant does not have access to the internet or is having difficulty completing the questionnaire, they can provide their answers via telephone to the study team who will manually input the participants' responses into the web-based application. Participants will be contacted by telephone to arrange the risk assessment appointment which will take place at the Nightingale Centre, part of the Manchester University NHS Foundation Trust. Before the appointment, participants will be sent a saliva sample collection tube in the post and asked to bring the saliva sample along to the appointment, which will be analysed for polygenic risk score (SNP313) and the presence of pathogenic variants in high and moderate-risk genes. At the appointment, participants will undergo low-dose mammography (two views of one breast only). Breast density will be calculated using a new technique called predicted visual assessment score (pVAS). pVAS is an automated method of



For peer review only - http://bmjopen.bmj.com/site/about/guidelines.xhtml

assessing mammograms using artificial intelligence techniques (22, 33). A risk feedback letter will be generated based on the answers participants give in their questionnaire, the results of genetic testing and mammographic density. The risk feedback letter will inform women that they are at “average” risk (< 3% 10-year risk) or “increased” risk (≥ 3% 10-year risk). Each letter will explain the implications of the risk result (see supplementary file 1). Participants identified as at increased risk will be offered an appointment at a FHRPC to discuss their risk result further with a breast clinician with expertise in risk assessment, screening and prevention. At this appointment, potential management options including earlier access to breast screening and risk-reducing medication will be discussed. All participants will receive their risk feedback letter within 16 weeks of the risk assessment appointment, along with leaflets providing additional detail on ways of reducing breast cancer risk, signs and symptoms of breast cancer and breast awareness. An updated risk feedback letter will be sent at the end of the study once the magnitude of risk associated with density is determined more accurately in this age group using all case control subjects. The timeline from the participant perspective is shown in Figure 1.

INSERT FIGURE 1: Timeline of feasibility study integrated with BCAN-RAY

***a. Health inequalities assessment***

GPs from participating general practices will extract self-reported ethnicity (where available) and deprivation information based on residential postcode for all women invited to take part in the BCAN-RAY study. They will provide this aggregated, non-identifiable data to the research team. No personally identifiable data will be shared with the research team as we predict the majority of women invited will not consent to the study. A member of the

For peer review only

research team will then extract the same information from the BCAN-RAY study database for all participants.

***b. Identification of potential harms and benefits***

Once participants have submitted the risk factors questionnaire on the web-based application, they will be directed to complete the baseline harms and benefits questionnaire on Qualtrics (https://www.qualtrics.com/uk/). If the baseline questionnaire has not been completed by the time a member of the study team rings the participant to arrange their risk assessment appointment, a reminder to do so will be enclosed with their appointment confirmation letter. Any remaining non-completers will be asked to complete the questionnaire online or via paper in the waiting room before their risk assessment appointment.

The same women will be asked to complete follow up questionnaires 6 weeks and 6 months after they have received their risk feedback. Women will be asked to input their unique BCAN-RAY study ID and their date of birth at the beginning of each questionnaire to ensure responses can be linked. Participants are able to request paper copies of the follow up questionnaires to be sent to them via post if preferred. The data recorded on paper copies of all questionnaires will be manually inputted into the Qualtrics platform by a member of the study team. If the follow up questionnaires have not been completed by two weeks after the initial invitations, a reminder to complete the questionnaire will be sent via email or letter.

***c. Understanding acceptability***



For peer review only - http://bmjopen.bmj.com/site/about/guidelines.xhtml

A purposive sample of average and increased risk women who complete the baseline and follow up questionnaires and have agreed to be contacted will be sent an invitation to participate in a semi-structured interview. Demographic characteristics and responses to questionnaires will guide sampling to allow variation in ethnicity, socioeconomic status, and knowledge and anxiety levels of participants. Average risk women will be invited for interview 1 month after receiving their risk feedback letter. Increased risk women will be invited for interview 6 months after receiving their risk feedback letter. This gives women at increased risk the chance to explore extra screening options or medications prior to the interview and minimises any influence participating in the interview may have on decision-making. We will aim to recruit up to 24 women to these interviews (up to 12 women in each risk category). If no response is received following the initial invitation, a second invitation will be sent approximately 3-4 weeks later.

Interviews will last approximately 40-60 minutes and will be conducted face-to-face or over the telephone according to each participant's preference. For face-to-face interviews, written consent will be obtained. For telephone interviews, verbal consent will be obtained over the telephone before the interview begins and recorded in a separate audio file. Interviews will be audio recorded and transcribed verbatim using an accredited transcription company. Participants will be compensated for their time with a £20 shopping voucher.

**Measures**

***a. Health inequalities assessment***



For peer review only - http://bmjopen.bmj.com/site/about/guidelines.xhtml

Residential postcode, a proxy measure of socioeconomic status, will be converted into deprivation deciles using the Index of Multiple Deprivation (IMD), a measure of relative deprivation for small areas in England (34). Where available, ethnicity data will be mapped onto the five high-level ethnic categories used in the 2021 Census for England (White, Asian/Asian British, Black/African/Caribbean/Black British, Mixed/Multiple, and Other ethnic group), in line with the current ethnicity harmonised standard (35). Missing data will be captured under two additional categories of refusal to provide information about ethnic group and no data available.

***b. Identification of potential harms and benefits***

The self-reported measures of potential harms and benefits of participation in breast cancer risk assessment to be completed by participants are shown in Table 2. A detailed description of each of these measures is provided in supplementary file 2. Supplementary file 3 contains a copy of each questionnaire.

**Table 2.** Self-reported measures to be assessed, at each of the three timepoints

| Baseline | 6 weeks post risk feedback | 6 months post risk feedback |
|---|---|---|
| State anxiety (36) | State anxiety (36) | State anxiety (36) |
| Cancer worry (37) | Cancer worry (37) | Cancer worry (37) |
| Risk perception (38) | Risk perception (38) | Risk perception (38) |



For peer review only - http://bmjopen.bmj.com/site/about/guidelines.xhtml

| | | |
|---|---|---|
| Attitudes towards risk assessment (39) | | Attitudes towards risk assessment (39) |
| | Knowledge[a] | |
| | Satisfaction with risk feedback information (40) | |
| | | Satisfaction with decision to participate in breast cancer risk assessment (41) |

[a]Assessed by a measure the research team has created as no validated measure available (see supplementary file 2 for more information about development of this measure)

### *c. Understanding acceptability*

Topic guide development was informed by the aims of the study and a review of the literature. An initial draft was developed by the lead author, a doctoral student in health psychology with qualitative health services research experience. Feedback on this draft was obtained from public contributors and members of the research team (DPF and JUS) who have research expertise in breast cancer and screening services, primary care and health services research, health psychology, and qualitative methods. The content and structure of the topic guide was revised in line with the feedback received. Participants will be asked about their experience of the risk assessment process including how acceptable they found it, their views on the materials developed for BCAN-RAY, and how the risk assessment process could be improved in terms of delivery/access and provision of information and support (see supplementary file 4). Furthermore, women will be asked to discuss any



For peer review only - http://bmjopen.bmj.com/site/about/guidelines.xhtml

actions they have considered and/or made as a result of participating in BCAN-RAY (e.g. lifestyle modifications, additional screening and risk-reducing medication).

## Data analysis

### *a. Health inequalities assessment*

The Chi-squared test will be used to compare uptake rates by ethnicity and socioeconomic status (assessed by IMD deciles) between women who participate in the BCAN-RAY study and women who decline participation. To ensure sufficient instances in each group, IMD deciles will be collapsed into quintiles and ethnicity will be collapsed into 6 subgroups (White, Asian, Black, Mixed or Multiple, Other and Missing).

### *b. Identification of potential harms and benefits*

The main analyses will focus on comparing the responses of the two groups of women provided with different risk estimates (average and increased) for outcomes assessed at multiple timepoints (i.e. anxiety, cancer worry, risk perceptions and attitudes towards breast cancer risk assessment). ANCOVA will be used, with baseline responses to the same variables, age and IMD deciles as covariates. Analyses will be conducted on all questionnaire measures at 6 weeks and 6 months, with the 6-month state anxiety measure being the primary outcome.

Measures administered at only one timepoint (knowledge, satisfaction with information received and satisfaction with decision to participate in breast cancer risk assessment) will be compared between the two groups of women provided with different risk estimates (average or increased). ANCOVA will be used, with age and IMD deciles as covariates.



For peer review only - http://bmjopen.bmj.com/site/about/guidelines.xhtml

All statistical tests will be two-sided and use a significance level of 5%. A “completer only” analysis strategy will be employed. If dropout levels are high, the a priori primary outcome (comparison of 6-month outcome scores between average and increased risk groups) will be repeated using a last occasion carried forward approach to missing data as a sensitivity analysis. Statistical analyses will be performed using SPSS.

***c. Understanding acceptability***

NVivo software will be used to organise the data. Data will be analysed using a manifest level approach to reflexive thematic analysis (42, 43). Thematic analysis involves examining qualitative data to produce themes that summarise and interpret patterns of results. Initial coding will be deductive based on the structured questions in the topic guide to address the objective of whether the BCAN-RAY approach is acceptable. Inductive methods will then be used to capture additional codes and context to ensure important aspects of the data are not missed. A critical realist approach will be adopted, with the researchers accepting that participants' accounts represent their perception of their reality, which is shaped by and embedded within their cultural context and language (44). An experiential orientation to data interpretation will be adopted that seeks to stay close to participants' meanings and capture these in ways that might be recognisable to them. The analysis will be conducted by the lead researcher with input from other members of the research team and public contributors.

**Sample size estimation**

***a. Health inequalities assessment***



For peer review only - http://bmjopen.bmj.com/site/about/guidelines.xhtml

The BCAN-RAY feasibility study aims to recruit approximately 750 women. Based on the results of the latest NHS GP Patient Survey in which 13-19% of those invited by post aged 25-44 responded (45), we conservatively expect a response rate of 10%. Therefore, approximately 7,500 invitations will be sent. If the response rate is lower than expected, more invitations will be sent until at least 750 women have been recruited. This approach will also yield 6,750 women who decline participation. Given the geographical spread of the general practices who have provisionally agreed to be involved in the study across different boroughs of Greater Manchester, we expect to recruit a socioeconomically diverse sample (see Table 3).

**Table 3.** Percentage of Lower Super Output Areas (LSOAs) in each deprivation decile across the boroughs of Greater Manchester involved in the BCAN-RAY study[a]

| | Location | | | | | |
|---|---|---|---|---|---|---|
| Deprivation decile[a] | Trafford | Manchester | Salford | Tameside | Rochdale | Stockport |
| 1-2 (most deprived) | 8.7% | 59.3% | 48.7% | 42.6% | 44.1% | 16.3% |
| 3-4 | 15.9% | 25.8% | 21.4% | 22.7% | 26.1% | 20% |
| 5-6 | 15.2% | 10.7% | 15.3% | 20.6% | 10.4% | 15.3% |
| 7-8 | 25.3% | 3.9% | 7.3% | 12.1% | 15% | 21.6% |
| 9-10 (least deprived) | 34.8% | 0.4% | 7.3% | 2.1% | 4.5% | 26.9% |



For peer review only - http://bmjopen.bmj.com/site/about/guidelines.xhtml

[a]Data sourced from an interactive map created by Greater Manchester Poverty Action (30)

[b]Assessed by the Index of Multiple Deprivation 2019 (34)

***b. Identification of potential harms and benefits***

The sample size for the BCAN-RAY study was based on providing sufficient power to be able to detect an effect of breast density, after adjustment for age and BMI. Therefore, a post hoc analysis was conducted to estimate achieved power with respect to the primary outcome of anxiety at 6 months. Assuming a two-tailed independent samples t-test and follow up questionnaire responses from 400 average risk women and 100 increased risk women, it is estimated that there will be approximately 76% power to detect a small, standardised difference of d = 0.3.

***c. Understanding acceptability***

The sample size for the BCAN-RAY study will provide more than sufficient numbers from which to recruit participants for the acceptability assessment. Whilst we anticipate including up to 24 participants in this component of the study (12 at average risk and 12 at increased risk), the decision to stop recruitment will be guided by the concept of 'information power'. The research team will reflect on the information richness of their dataset throughout data collection to determine when sufficient data has been collected to answer the research question (46).

**Public involvement**



For peer review only - http://bmjopen.bmj.com/site/about/guidelines.xhtml

A public involvement group of 11 women aged 30-39 years was established in September 2021 to inform the development of research aimed at identifying young women at increased risk of breast cancer including the BCAN-RAY study. Five women reviewed the study documentation (participant information sheet, consent form, study invite letter, risk feedback letters, baseline and follow up questionnaires, and interview topic guide). The content and structure of documentation was revised in line with the feedback received. Changes included the removal of one question from the knowledge measure as it overlapped considerably with the content of one of the other questions and the addition of breast cancer charity contact information to risk feedback letters. We will continue to involve members of the public involvement group in subsequent stages of the research cycle including analysis of interview data and dissemination.

**Ethics and dissemination**

This study was approved by the North West - Greater Manchester West Research Ethics Committee (reference: 22/NW/0268). The study will be performed in accordance with the Declaration of Helsinki, Good Clinical Practice principles and relevant regulations. All participants in BCAN-RAY complete written consent online. All participants will provide informed consent (written if face-to-face, verbal if over telephone) prior to taking part in an interview. Quantitative study data will be tracked via participant study IDs. Identifying information will be removed from the interview transcripts and participants will be assigned pseudonyms.

We will disseminate our findings through peer-reviewed journals, conference presentations and charitable organisations. At the time of consent for both the BCAN-RAY study and an



For peer review only - http://bmjopen.bmj.com/site/about/guidelines.xhtml

interview, participants will be asked to indicate whether they wish to receive a summary of findings. A written lay summary will be produced and sent to those who opt to receive this.

## Discussion

The present research aims to provide evidence on the feasibility of a strategy to offer breast cancer risk assessment based on family history, phenotypic risk factors, polygenic risk and mammographic density to women aged 30-39 years. It will provide information about uptake rates, potential harms and benefits of participation, and the acceptability of the risk assessment strategy including novel insight into the experience of low-dose mammography amongst a population of women not known to be at increased risk of breast cancer.

One key issue that the present research does not cover relates to whether breast cancer risk assessment in younger women is acceptable to healthcare professionals involved in its delivery, which is recognised as an important component of feasibility (47). We have interviewed and conducted focus groups with primary care professionals to understand their views on involvement in breast cancer risk assessment and management and analysis is ongoing. However, as the optimal strategy for implementation remains unclear, it is not yet known who would be responsible for the delivery of risk assessment. Future research investigating alternative strategies for implementation ought to consider the views of healthcare personnel involved in delivery to establish likely effects on the healthcare system when implementing risk assessment.

The study will provide valuable information about whether a primary care co-ordinated invitation process is successful at engaging women from diverse socioeconomic and ethnic backgrounds thereby informing the need to consider and evaluate alternative invitation



For peer review only - http://bmjopen.bmj.com/site/about/guidelines.xhtml

methods prior to further implementation. Furthermore, findings will provide information about the likely harms and benefits of participation in breast cancer risk assessment and identify modifications needed to the risk assessment strategy to increase engagement and uptake in future implementation studies.

Key feasibility issues for implementing risk-stratified screening into routine breast cancer screening have now been identified. The present study provides an important first step in assessing the feasibility of introducing comprehensive breast cancer risk assessment for younger women to enable those identified as being at increased risk access to screening and preventive strategies in the absence of a family history of breast cancer.

## Declarations

### Author Contributions

The BCAN-RAY study was conceived and designed and is being led by SJH and DGE. Funding for BCAN-RAY was led by SJH and DGE, with input from JAU-S and DPF. The feasibility study and participant documentation were designed by SH, SJH, JAU-S and DPF. SH co-ordinated the involvement of public contributors. The present article was drafted by SH. DPF, SJH, LG, JAU-S and DGE provided feedback on versions of the manuscript. All authors read and approved the final manuscript.

### Competing interests

The authors declare that they have no competing interests.



For peer review only - http://bmjopen.bmj.com/site/about/guidelines.xhtml

**Funding**

This study is sponsored by Manchester University NHS Foundation Trust and funded by grants from Cancer Research UK Alliance for Cancer Early Detection (ref: EDDAMC-2021\100003) and The Christie Charity. The web-based application was developed through charitable donations from the Shine Bright Foundation and Tony Thornley. The low-dose mammogram was developed with a grant from the Medical Research Council's Confidence in Concept funding scheme (2018/19). SH is funded by a Manchester Cancer Research Centre PhD studentship. DPF, DGE and SJH are supported by the NIHR Manchester Biomedical Research Centre (IS-BRC-1215-20007 and NIHR203308). JAU-S is funded by an Advanced Fellowship from the National Institute for Health and Social Care Research (NIHR300861). The views expressed are those of the authors and not necessarily those of the NHS, the NIHR or the Department of Health and Social Care. These funding sources had no role in the design of this study and will not have any role during its execution, analyses, interpretation of the data, or decision to submit results.

**Acknowledgements**

We would like to thank Stephanie Archer who helped with the developmental work. We also gratefully acknowledge the contributions of our public involvement group.

## References

1. Heer E, Harper A, Escandor N, Sung H, McCormack V, Fidler-Benaoudia MM. Global burden and trends in premenopausal and postmenopausal breast cancer: a population-based study. Lancet Glob Health. 2020;8(8):e1027-e37.

2. Sung H, Ferlay J, Siegel RL, Laversanne M, Soerjomataram I, Jemal A, et al. Global cancer statistics 2020: GLOBOCAN estimates of incidence and mortality worldwide for 36 cancers in 185 countries. CA Cancer J Clin. 2021;71(3):209-49.

3. Copson ER, Maishman TC, Tapper WJ, Cutress RI, Greville-Heygate S, Altman DG, et al. Germline BRCA mutation and outcome in young-onset breast cancer (POSH): a prospective cohort study. Lancet Oncol. 2018;19(2):169-80.

4. Assi HA, Khoury KE, Dbouk H, Khalil LE, Mouhieddine TH, El Saghir NS. Epidemiology and prognosis of breast cancer in young women. J Thorac Dis. 2013;5(Suppl 1):S2-8.

5. Bardia A, Hurvitz S. Targeted therapy for premenopausal women with HR+, HER2− advanced breast cancer: focus on special considerations and latest advances. Clin Cancer Res. 2018;24(21):5206-18.

6. Lian W, Fu F, Lin Y, Lu M, Chen B, Yang P, et al. The impact of young age for prognosis by subtype in women with early breast cancer. Sci Rep. 2017;7(1):11625.

7. Office for National Statistics. Deaths registered in England and Wales: 2021. 2022. www.ons.gov.uk/peoplepopulationandcommunity/birthsdeathsandmarriages/deaths/bulletins/deathsregistrationsummarytables/2021. Accessed 19 Apr 2023.

8. Kudela E, Samec M, Kubatka P, Nachajova M, Laucekova Z, Liskova A, et al. Breast cancer in young women: status quo and advanced disease management by a predictive, preventive, and personalized approach. Cancers. 2019;11(11):1791.



For peer review only - http://bmjopen.bmj.com/site/about/guidelines.xhtml

9. Evans D, Brentnall AR, Harvie M, Dawe S, Sergeant JC, Stavrinos P, et al. Breast cancer risk in young women in the National Breast Screening Programme: implications for applying NICE guidelines for additional screening and chemoprevention. Cancer Prev Res. 2014;7(10):993-1001.

10. National Institute for Health and Care Excellence [NICE]. Familial breast cancer: classification, care and managing breast cancer and related risks in people with a family history of breast cancer (updated Nov 2019). 2013. https://www.nice.org.uk/guidance/cg164/chapter/Recommendations. Accessed 19 Apr 2023.

11. Eccles BK, Copson ER, Cutress RI, Maishman T, Altman DG, Simmonds P, et al. Family history and outcome of young patients with breast cancer in the UK (POSH study). Br J Surg. 2015;102(8):924-35.

12. Dite GS, MacInnis RJ, Bickerstaffe A, Dowty JG, Allman R, Apicella C, et al. Breast cancer risk prediction using clinical models and 77 independent risk-associated SNPs for women aged under 50 years: Australian breast cancer family registry. Cancer Epidemiol Biomarkers Prev. 2016;25(2):359-65.

13. Evans D, Harkness EF, Brentnall AR, van Veen EM, Astley SM, Byers H, et al. Breast cancer pathology and stage are better predicted by risk stratification models that include mammographic density and common genetic variants. Breast Cancer Res Treat. 2019;176(1):141-8.

14. Hurson AN, Pal Choudhury P, Gao C, Hüsing A, Eriksson M, Shi M, et al. Prospective evaluation of a breast-cancer risk model integrating classical risk factors and polygenic risk in 15 cohorts from six countries. Int J Epidemiol. 2021;50(6):1897-911.

For peer review only



For peer review only - http://bmjopen.bmj.com/site/about/guidelines.xhtml

15. Vilmun BM, Vejborg I, Lynge E, Lillholm M, Nielsen M, Nielsen MB, et al. Impact of adding breast density to breast cancer risk models: a systematic review. Eur J Radiol. 2020;127:109019.

16. Evans DGR, Donnelly LS, Harkness EF, Astley SM, Stavrinos P, Dawe S, et al. Breast cancer risk feedback to women in the UK NHS breast screening population. Br J Cancer. 2016;114(9):1045-52.

17. van Veen EM, Brentnall AR, Byers H, Harkness EF, Astley SM, Sampson S, et al. Use of single-nucleotide polymorphisms and mammographic density plus classic risk factors for breast cancer risk prediction. JAMA Oncol. 2018;4(4):476-82.

18. Esserman LJ, Anton-Culver H, Borowsky A, Brain S, Cink T, Crawford B, et al. The WISDOM Study: breaking the deadlock in the breast cancer screening debate. NPJ Breast Cancer. 2017;3(1):34.

19. My Personalized Breast Screening (MyPeBS). ClinicalTrials.gov identifier: NCT03672331. 2018. https://clinicaltrials.gov/ct2/show/NCT03672331. Accessed 19 Apr 2023.

20. Usher-Smith JA, Hindmarch S, French DP, Tischkowitz M, Moorthie S, Walter FM, et al. Proactive breast cancer risk assessment in primary care: a review based on the principles of screening. Br J Cancer. 2023;128:1636-46.

21. Breast CANcer Risk Assessment in Younger Women: BCAN-RAY (BCAN-RAY). ClinicalTrials.gov identifier: NCT04336904. 2022. https://clinicaltrials.gov/ct2/show/NCT05305963. Accessed 19 Apr 2023.

22. Squires S, Ionescu G, Harkness E, Mackenzie A, Evans D, Maxwell A, et al. Automatic density prediction in low dose mammography. Proc. SPIE 11513, 15th International Workshop on Breast Imaging; 2020. Available from: https://doi.org/10.1117/12.2564714.



For peer review only - http://bmjopen.bmj.com/site/about/guidelines.xhtml

23. Skivington K, Matthews L, Simpson SA, Craig P, Baird J, Blazeby JM, et al. A new framework for developing and evaluating complex interventions: update of Medical Research Council guidance. BMJ. 2021;374:n2061.

24. Evans DGR, McWilliams L, Astley S, Brentnall AR, Cuzick J, Dobrashian R, et al. Quantifying the effects of risk-stratified breast cancer screening when delivered in real time as routine practice versus usual screening: the BC-Predict non-randomised controlled study (NCT04359420). Br J Cancer. 2023;128:2063-71.

25. U.S. Preventive Services Task Force [USPSTF]. Breast Cancer: Screening (Draft Recommendation Statement). 2023. https://www.uspreventiveservicestaskforce.org/uspstf/draft-recommendation/breast-cancer-screening-adults. Accessed 19 Apr 2023.

26. French DP, McWilliams L, Bowers S, Woof VG, Harrison F, Ruane H, et al. Psychological impact of risk-stratified screening as part of the NHS Breast Screening Programme: multi-site non-randomised comparison of BC-Predict versus usual screening (NCT04359420). Br J Cancer. 2023;128:1548-58.

27. French DP, Southworth J, Howell A, Harvie M, Stavrinos P, Watterson D, et al. Psychological impact of providing women with personalised 10-year breast cancer risk estimates. Br J Cancer. 2018;118(12):1648-57.

28. Hindmarch S, Gorman L, Hawkes RE, Howell SJ, French DP. Optimising the delivery of breast cancer risk assessment for women aged 30–39 years: A qualitative study of women's views. Womens Health. 2023;19.

29. Green CA, Duan N, Gibbons RD, Hoagwood KE, Palinkas LA, Wisdom JP. Approaches to mixed methods dissemination and implementation research: methods, strengths, caveats, and opportunities. Adm Policy Ment. 2015;42(5):508-23.

For peer review only

30. Greater Manchester Poverty Action. Deprivation at a neighbourhood level. 2023 https://www.gmpovertyaction.org/pm2022-imd/. Accessed 22 May 2023.

31. Office for National Statistics. Ethnic group, England and Wales: Census 2021. 2022. https://www.ons.gov.uk/peoplepopulationandcommunity/culturalidentity/ethnicity/bulletins/ethnicgroupenglandandwales/census2021#how-ethnic-composition-varied-across-england-and-wales. Accessed 22 May 2023.

32. Tyrer J, Duffy SW, Cuzick J. A breast cancer prediction model incorporating familial and personal risk factors. Stat Med. 2004;23(7):1111-30.

33. Astley SM, Harkness EF, Sergeant JC, Warwick J, Stavrinos P, Warren R, et al. A comparison of five methods of measuring mammographic density: a case-control study. Breast Cancer Res. 2018;20(1):10.

34. Ministry of Housing, Communities, Local Government. English indices of deprivation 2019. 2019. https://imd-by-postcode.opendatacommunities.org/imd/2019. Accessed 22 May 2023.

35. Government Statistical Service. Ethnicity harmonised standard. 2011. https://analysisfunction.civilservice.gov.uk/policy-store/ethnicity-harmonised-standard/. Accessed 22 May 2023.

36. Marteau TM, Bekker H. The development of a six-item short-form of the state scale of the Spielberger State—Trait Anxiety Inventory (STAI). Br J Clin Psychol. 1992;31(3):301-6.

37. Lerman C, Trock B, Rimer BK, Jepson C, Brody D, Boyce A. Psychological side effects of breast cancer screening. Health Psychol. 1991;10(4):259-67.

38. Weinstein ND. What does it mean to understand a risk? Evaluating risk comprehension. J Natl Cancer Inst Monographs. 1999;1999(25):15-20.



For peer review only - http://bmjopen.bmj.com/site/about/guidelines.xhtml

39. Ajzen I. Understanding attitudes and predicting social behavior. Englewood Cliffs, New Jersey: Prentice Hall; 1980.

40. French DP, Maissi E, Marteau TM. The psychological costs of inadequate cervical smear test results: three-month follow-up. Psychooncology. 2006;15(6):498-508.

41. Brehaut JC, O'Connor AM, Wood TJ, Hack TF, Siminoff L, Gordon E, et al. Validation of a decision regret scale. Med Decis Making. 2003;23(4):281-92.

42. Braun V, Clarke V. Using thematic analysis in psychology. Qual Res Psychol. 2006;3(2):77-101.

43. Braun V, Clarke V. Reflecting on reflexive thematic analysis. Qual Res Sport Exerc Health. 2019;11(4):589-97.

44. Pilgrim D. Some implications of critical realism for mental health research. Soc Theory Health. 2014;12(1):1-21.

45. NHS England. GP Patient Survey 2022: Technical Annex. 2022. https://www.gp-patient.co.uk/surveysandreports. Accessed 22 May 2023.

46. Malterud K, Siersma VD, Guassora AD. Sample size in qualitative interview studies: guided by information power. Qual Health Res. 2016;26(13):1753-60.

47. Skivington K, Matthews L, Simpson S, Craig P, Baird J, JM B, et al. Framework for the development and evaluation of complex interventions: gap analysis, workshop and consultation-informed update. Health Technol Assess. 2021;25(57):1-132.

For peer review only

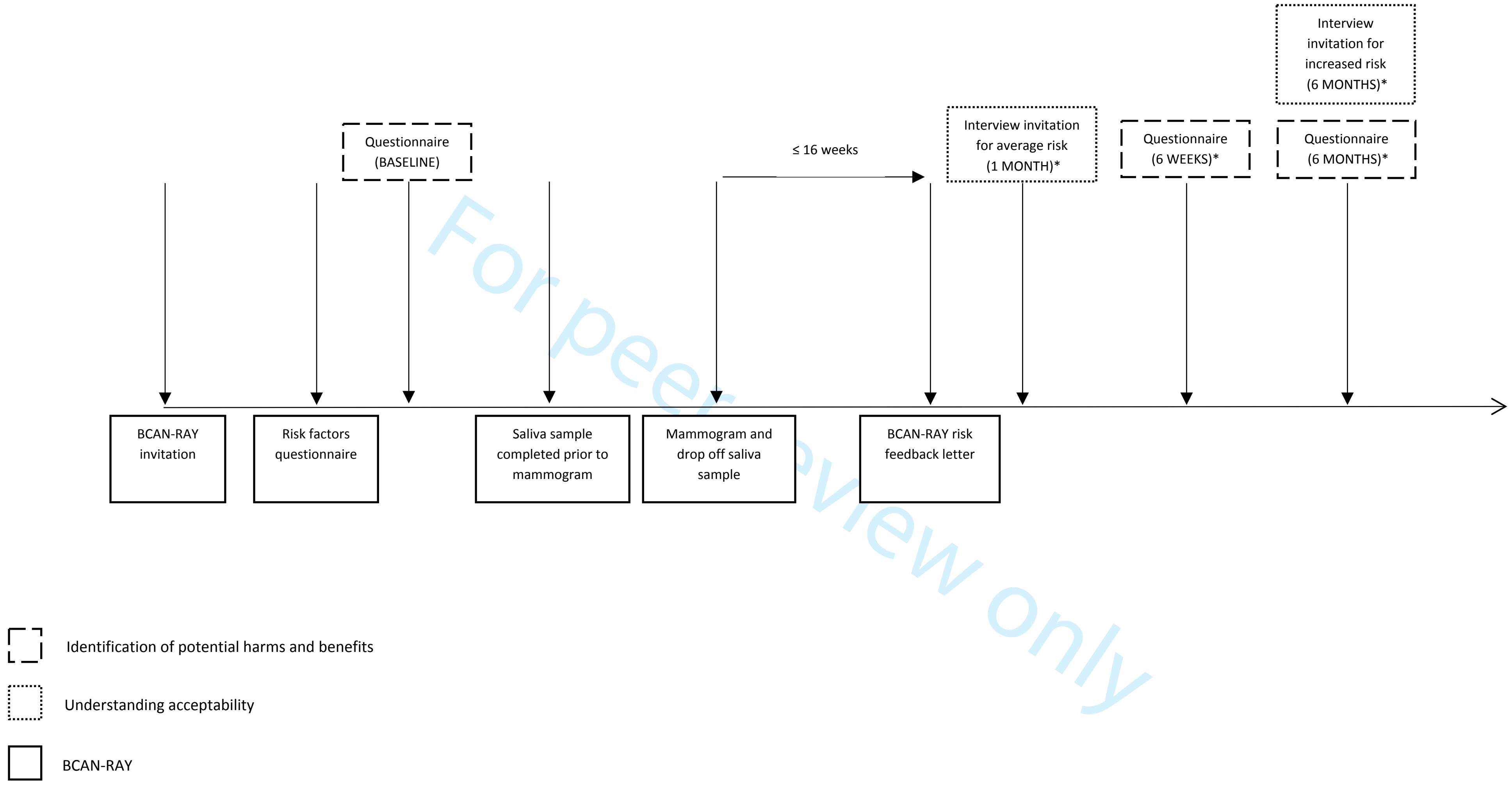


*Duration from risk feedback letter

For peer review only

**Supplementary file 1.** BCAN-RAY risk feedback letters (average, increased)

**INSERT LOGOS**

Nightingale Centre, Wythenshawe Hospital
Manchester University NHS Foundation Trust
Southmoor Road
Manchester
M23 9LT
**Tel: INSERT NUMBER**

**INSERT PARTICIPANT NAME**
**INSERT ADDRESS**
**INSERT ADDRESS**
**INSERT ADDRESS**

**INSERT POSTCODE**

Date: **INSERT DATE**

Dear **[INSERT NAME]**,

**RE: BCAN-RAY Study**
**NHS number: INSERT**

Thank you for taking part in the BCAN-RAY study. This is your first risk feedback letter. A second letter will follow when the study is complete for all women (probably in 2025). It is possible that the second letter may change your risk level.

We have calculated your risk of developing breast cancer in the next 10 years from the following information collected in this study:

- Breast cancer risk factors as assessed from the information you provided on the risk factor questionnaire
- Breast density (the amount of tissue in your breast that is not fat) as assessed from your mammogram
- DNA as assessed from your saliva (spit) sample

Your risk of developing breast cancer in the next 10 years was calculated to be:

**Average for the population – that is less than 3 in 100 chance of developing breast cancer in the next 10 years.**

More detailed information about your risk result is given in the enclosed document. This information is also available on the study web-based application, which can be accessed by scanning this QR code:

**INSERT QR CODE FOR WEB BASED APPLICATION**

We also confirm that no pathological variants (mutations) were identified in the 9 risk genes analysed in your saliva sample DNA.

For peer review only

**Further information and support resources**

There are things that all women can do to reduce their risk of breast cancer, such as maintaining a healthy weight through diet and exercise and limiting alcohol intake. More information on the ways to reduce your risk is provided in the accompanying leaflet. It is also important to regularly check your breasts and report anything new or unusual to a GP. A guide explaining how to check your breasts is enclosed.

Additionally, you may find the following sources of information and support useful if you have any breast health concerns.

**CoppaFeel!**

Website: https://coppafeel.org/

**Breast Cancer Now**

Website: https://breastcancernow.org/

They have a section where you can ask any questions you have relating to breast health:

https://forum.breastcancernow.org/t5/Ask-Our-Nurses/ct-p/Asknurses

They also offer a free, confidential helpline to answer questions about breast cancer or breast health – 0808 800 6000 (Text relay prefix – 18001)

**Should you have any questions about the study please get in touch with the study team on INSERT NUMBER**.

Yours sincerely,

**INSERT SIGNATURE**
**INSERT NAME**

For peer review only

INSERT LOGOS

Nightingale Centre, Wythenshawe Hospital
Manchester University NHS Foundation Trust
Southmoor Road
Manchester
M23 9LT
**Tel:** INSERT NUMBER

**INSERT PARTICIPANT NAME**
**INSERT ADDRESS**
**INSERT ADDRESS**
**INSERT ADDRESS**

**INSERT POSTCODE**

Date: **INSERT DATE**

Dear **[INSERT NAME]**,

**RE: BCAN-RAY Study**
**NHS number: INSERT**

Thank you for taking part in the BCAN-RAY study. This is your first risk feedback letter.

A second letter will follow when the study is complete for all women (probably in 2025). It is possible that the second letter may change your risk level.

**Your result:**

You are at **increased** risk of breast cancer

This means that you are more likely to develop breast cancer than other women your age in the general population.

The details of your 10 year risk and lifetime risk of breast cancer compared to the general population are provided in the attached document and are also available on the study web-based application, which can be accessed by scanning this QR code:

**INSERT QR CODE FOR WEB BASED APPLICATION**

The factors that may have increased your personal risk were:

- Breast cancer risk factors as assessed from the information you provided on the risk factor questionnaire
- Breast density (the amount of tissue in your breast that is not fat) as assessed from your mammogram
- DNA as assessed from your saliva (spit) sample

At this level of risk you will be eligible to start breast screening earlier than the general population and will have access to breast cancer risk reducing approaches.

For peer review only

**Gene mutation search**

We did not identify a pathological variant (mutation) in any of the 9 risk genes tested.

OR

We have also identified a pathological variant (mutation) in one of the 9 risk genes tested. We would like to give you the opportunity to discuss the potential implications of this for yourself and your family in more detail and the planned risk review appointment (see below) will be with a geneticist (a doctor who specialises in gene mutations and what they mean for families).

**Risk review appointment**

We would like to offer you a face-to-face appointment at the Family History Risk and Prevention Clinic at The Nightingale Centre to discuss your risk result further. During this appointment, your breast cancer risk will be explained to you along with information about additional breast screening and when this can begin in addition to ways to reduce your risk.

This appointment is part of NHS care and not part of the study itself. As such, a referral into the clinic will be made by your GP and an appointment will be arranged. This should be within 8-12 weeks so if you have not received an appointment 8 weeks after receiving your risk result, please contact the Nightingale team on **INSERT NUMBER**.

**Further information and support resources**

There are things that all women can do to reduce their risk of breast cancer, such as maintaining a healthy weight through diet and exercise and limiting alcohol intake. More information on the ways to reduce your risk is provided in the accompanying leaflet. It is also important to regularly check your breasts and report anything new or unusual to a GP. A guide explaining how to check your breasts is enclosed.

Additionally, you may find the following sources of information and support useful if you have any breast health concerns.

**CoppaFeel!**

Website: https://coppafeel.org/

**Breast Cancer Now**

Website: https://breastcancernow.org/

They have a section where you can ask any questions you have relating to breast health:

https://forum.breastcancernow.org/t5/Ask-Our-Nurses/ct-p/Asknurses

They also offer a free, confidential helpline to answer questions about breast cancer or breast health – 0808 800 6000 (Text relay prefix – 18001)

**Should you have any questions about the study please get in touch with the study team on INSERT NUMBER.**

Yours sincerely,

**INSERT SIGNATURE**
**INSERT NAME**

For peer review only

**Supplementary file 3.** Participant questionnaires (baseline, 6 weeks post risk feedback and 6 months post risk feedback)

# **B**reast **CAN**cer – **R**isk Assessment in **Y**oung Women **(BCAN-RAY):**
# Acceptability survey (baseline)

**Please enter your unique identifier and date of birth. Your unique identifier can be found on your study invite letter.**

**Unique study identifier:**

**Date of birth:**

For peer review only

**SECTION A – YOUR MENTAL WELL-BEING**
A number of statements which people have used to describe how they feel are given below. Please read each of the 6 statements and then circle the most appropriate number below the statement to indicate how you feel right now, at this moment. There are no right or wrong answers. Do not spend too much time on any one statement but give the answer which seems to describe your present feelings best.

**A1** **I feel calm**

| Not at all | Somewhat | Moderately | Very much |
|---|---|---|---|
| 1 | 2 | 3 | 4 |

**A2** **I am tense**

| Not at all | Somewhat | Moderately | Very much |
|---|---|---|---|
| 1 | 2 | 3 | 4 |

**A3** **I feel upset**

| Not at all | Somewhat | Moderately | Very much |
|---|---|---|---|
| 1 | 2 | 3 | 4 |

**A4** **I am relaxed**

| Not at all | Somewhat | Moderately | Very much |
|---|---|---|---|
| 1 | 2 | 3 | 4 |

**A5** **I feel content**

| Not at all | Somewhat | Moderately | Very much |
|---|---|---|---|
| 1 | 2 | 3 | 4 |

**A6** **I am worried**

| Not at all | Somewhat | Moderately | Very much |
|---|---|---|---|
| 1 | 2 | 3 | 4 |

For peer review only

**SECTION B – YOUR WORRIES ABOUT DEVELOPING BREAST CANCER**
Please read the statements below and circle the number below each statement that best indicates your current level of worry about getting breast cancer someday:

**B1** **How often have you thought about your chances of getting breast cancer?**

| Never | Rarely | Sometimes | Almost all the time |
|---|---|---|---|
| 1 | 2 | 3 | 4 |

**B2** **How often have these thoughts affected your mood?**

| Never | Rarely | Sometimes | Almost all the time |
|---|---|---|---|
| 1 | 2 | 3 | 4 |

**B3** **How often have these thoughts interfered with your ability to do daily activities?**

| Never | Rarely | Sometimes | Almost all the time |
|---|---|---|---|
| 1 | 2 | 3 | 4 |

**B4** **How concerned are you about the possibility of getting breast cancer one day?**

| Not at all | A little | Somewhat | A lot |
|---|---|---|---|
| 1 | 2 | 3 | 4 |

**B5** **How often do you worry about developing breast cancer?**

| Never | Rarely | Sometimes | Almost all the time |
|---|---|---|---|
| 1 | 2 | 3 | 4 |

**B6** **How much of a problem is this worry?**

| Not at all | A little | Somewhat | A lot |
|---|---|---|---|
| 1 | 2 | 3 | 4 |

For peer review only

**SECTION C – YOUR PERCEPTION OF BREAST CANCER RISK**
Please tick **ONE** of the statements below that best describes your breast cancer risk in relation to other women of a similar age:

**C1** **Compared to other women my age, I believe my risk of developing breast cancer in the next 10 years is…**

- ❑ Much higher
- ❑ A bit higher
- ❑ About the same
- ❑ A bit lower
- ❑ Much lower

**SECTION D – YOUR ATTITUDES TOWARD BREAST CANCER RISK ASSESSMENT**
Please read the statement and items below and circle the number that best indicates how you feel about participating in breast cancer risk assessment right now, at this moment:

**D1** **Taking part in breast cancer risk assessment will be…**

| Entirely good | Mainly good | Neither good nor bad | Mainly bad | Entirely bad |
|---|---|---|---|---|
| 1 | 2 | 3 | 4 | 5 |
| Entirely beneficial | Mainly beneficial | Neither beneficial nor harmful | Mainly harmful | Entirely harmful |
| 1 | 2 | 3 | 4 | 5 |
| Entirely important | Mainly important | Neither important nor unimportant | Mainly unimportant | Entirely unimportant |
| 1 | 2 | 3 | 4 | 5 |

For peer review only

**SECTION E – INTEREST IN INTERVIEW**
We would like to hear more about your experience of participating in breast cancer risk assessment as part of the BCAN-RAY study. Please tick one box to indicate whether you are happy to be contacted about participating in an interview (over the phone or face-to-face).

**E1** **I am happy to be contacted about participating in an interview following receipt of my risk results**

YES ❑ NO ❑

***Thank you for completing this questionnaire.***
***Please return your completed questionnaire to the study team in the pre-paid envelope provided.***

**Sources of information and support**

You may find some of the following sources of information and support useful if you have any concerns about breast health.

**CoppaFeel!**

Website: https://coppafeel.org/

They have a section that provides guidance on checking your breasts:
https://self-checkout.coppafeel.org/onboarding

**Breast Cancer Now**

Website: https://breastcancernow.org/

They have a section where you can ask any questions you have relating to breast health:

https://forum.breastcancernow.org/t5/Ask-Our-Nurses/ct-p/Asknurses

They also offer a free, confidential helpline to answer questions about breast cancer or breast health – 0808 800 6000 (Text relay prefix – 18001)

For peer review

# **B**reast **CAN**cer **– R**isk Assessment in **Y**oung Women **(BCAN-RAY):**

# Acceptability survey (6 weeks post risk feedback)

**Please enter your unique identifier and date of birth. Your unique identifier can be found on your study invite letter.**

**Unique study identifier:**

**Date of birth:**

For peer review only

**SECTION A – YOUR MENTAL WELL-BEING**

A number of statements which people have used to describe how they feel are given below. Please read each of the 6 statements and then circle the most appropriate number below the statement to indicate how you feel right now, at this moment. There are no right or wrong answers. Do not spend too much time on any one statement but give the answer which seems to describe your present feelings best.

**A1** **I feel calm**

| Not at all | Somewhat | Moderately | Very much |
|---|---|---|---|
| 1 | 2 | 3 | 4 |

**A2** **I am tense**

| Not at all | Somewhat | Moderately | Very much |
|---|---|---|---|
| 1 | 2 | 3 | 4 |

**A3** **I feel upset**

| Not at all | Somewhat | Moderately | Very much |
|---|---|---|---|
| 1 | 2 | 3 | 4 |

**A4** **I am relaxed**

| Not at all | Somewhat | Moderately | Very much |
|---|---|---|---|
| 1 | 2 | 3 | 4 |

**A5** **I feel content**

| Not at all | Somewhat | Moderately | Very much |
|---|---|---|---|
| 1 | 2 | 3 | 4 |

**A6** **I am worried**

| Not at all | Somewhat | Moderately | Very much |
|---|---|---|---|
| 1 | 2 | 3 | 4 |

For peer review only

**SECTION B – YOUR WORRIES ABOUT DEVELOPING BREAST CANCER**
Please read the statements below and circle the number below each statement that best indicates your current level of worry about getting breast cancer someday:

**B1** **How often have you thought about your chances of getting breast cancer?**

| Never | Rarely | Sometimes | Almost all the time |
|---|---|---|---|
| 1 | 2 | 3 | 4 |

**B2** **How often have these thoughts affected your mood?**

| Never | Rarely | Sometimes | Almost all the time |
|---|---|---|---|
| 1 | 2 | 3 | 4 |

**B3** **How often have these thoughts interfered with your ability to do daily activities?**

| Never | Rarely | Sometimes | Almost all the time |
|---|---|---|---|
| 1 | 2 | 3 | 4 |

**B4** **How concerned are you about the possibility of getting breast cancer one day?**

| Not at all | A little | Somewhat | A lot |
|---|---|---|---|
| 1 | 2 | 3 | 4 |

**B5** **How often do you worry about developing breast cancer?**

| Never | Rarely | Sometimes | Almost all the time |
|---|---|---|---|
| 1 | 2 | 3 | 4 |

**B6** **How much of a problem is this worry?**

| Not at all | A little | Somewhat | A lot |
|---|---|---|---|
| 1 | 2 | 3 | 4 |

For peer review only

**SECTION C – YOUR PERCEPTION OF BREAST CANCER RISK**
Please tick **ONE** of the statements below that best describes your breast cancer risk in relation to other women of a similar age:

**C1** **Compared to other women my age, I believe my risk of developing breast cancer in the next 10 years is…**

- ❑ Much higher
- ❑ A bit higher
- ❑ About the same
- ❑ A bit lower
- ❑ Much lower

**SECTION D – YOUR BREAST CANCER RISK KNOWLEDGE**
Please read the statement below and then circle the most appropriate number below the statement to indicate how informed you feel about your breast cancer risk at this moment:

**D1** **How informed do you feel about your breast cancer risk?**

| Very well informed | Quite well informed | Quite uninformed | Not very informed at all |
|---|---|---|---|
| 1 | 2 | 3 | 4 |

For peer review only

**SECTION E – YOUR KNOWLEDGE**

For each question please place **ONE** tick in the box that corresponds with your knowledge/understanding of breast cancer risk assessment being offered in the BCAN-RAY study.

**E1** **Who are the intended participants of breast cancer risk assessment in the BCAN-RAY study?**

- ❑ Women who have been told by a healthcare professional that they have a strong family history of breast cancer
- ❑ Women who have not been told by a healthcare professional that they have a strong family history of breast cancer

**E2** **What is the purpose of the low dose mammogram in the BCAN-RAY study?**

- ❑ To assess breast density (the amount of tissue in your breast that is not fat)
- ❑ To detect breast cancer

**E3** **Who will be given the opportunity to discuss additional breast screening and risk reducing measures with a clinician in the BCAN-RAY study?**

- ❑ Only women identified as being at increased risk of breast cancer
- ❑ All women who participate in the study

For peer review only

**SECTION F – YOUR PERCEPTIONS OF THE BREAST CANCER INFORMATION ENCLOSED WITH YOUR RISK FEEDBACK**

Thinking about the letter and leaflets you received when you were provided with your risk of developing breast cancer in the next 10 years, please read each statement and then circle the most appropriate number below the statement to indicate how you feel about the information *(please circle **only one number**).*

**F1** **I feel well informed about my breast cancer risk.**

| Strongly agree | Agree | Somewhat agree | Undecided | Disagree somewhat | Disagree | Strongly disagree |
|---|---|---|---|---|---|---|
| 1 | 2 | 3 | 4 | 5 | 6 | 7 |

**F2** **I feel satisfied with the amount of information I have been given.**

| Strongly agree | Agree | Somewhat agree | Undecided | Disagree somewhat | Disagree | Strongly disagree |
|---|---|---|---|---|---|---|
| 1 | 2 | 3 | 4 | 5 | 6 | 7 |

**F3** **I am confused by the information I have been given.**

| Strongly agree | Agree | Somewhat agree | Undecided | Disagree somewhat | Disagree | Strongly disagree |
|---|---|---|---|---|---|---|
| 1 | 2 | 3 | 4 | 5 | 6 | 7 |

**F4** **The information was clear.**

| Strongly agree | Agree | Somewhat agree | Undecided | Disagree somewhat | Disagree | Strongly disagree |
|---|---|---|---|---|---|---|
| 1 | 2 | 3 | 4 | 5 | 6 | 7 |

***Thank you for completing this questionnaire.***
***Please return your completed questionnaire to the study team in the pre-paid envelope provided.***

For peer review only

## Sources of information and support

You may find some of the following sources of information and support useful if you have any concerns about breast health.

**CoppaFeel!**

Website: https://coppafeel.org/

They have a section that provides guidance on checking your breasts:
https://self-checkout.coppafeel.org/onboarding

**Breast Cancer Now**

Website: https://breastcancernow.org/

They have a section where you can ask any questions you have relating to breast health:

https://forum.breastcancernow.org/t5/Ask-Our-Nurses/ct-p/Asknurses

They also offer a free, confidential helpline to answer questions about breast cancer or breast health – 0808 800 6000 (Text relay prefix – 18001)

For peer review

# **B**reast **CAN**cer **– R**isk Assessment in **Y**oung Women **(BCAN-RAY):** Acceptability survey (6 months post risk feedback)

For peer review only

**Please enter your unique identifier and date of birth. Your unique identifier can be found on your study invite letter.**

**Unique study identifier:**

**Date of birth:**

**SECTION A – YOUR MENTAL WELL-BEING**

A number of statements which people have used to describe how they feel are given below. Please read each of the 6 statements and then circle the most appropriate number below the statement to indicate how you feel right now, at this moment. There are no right or wrong answers. Do not spend too much time on any one statement but give the answer which seems to describe your present feelings best.

**A1** **I feel calm**

| Not at all | Somewhat | Moderately | Very much |
|---|---|---|---|
| 1 | 2 | 3 | 4 |

**A2** **I am tense**

| Not at all | Somewhat | Moderately | Very much |
|---|---|---|---|
| 1 | 2 | 3 | 4 |

**A3** **I feel upset**

| Not at all | Somewhat | Moderately | Very much |
|---|---|---|---|
| 1 | 2 | 3 | 4 |

**A4** **I am relaxed**

| Not at all | Somewhat | Moderately | Very much |
|---|---|---|---|
| 1 | 2 | 3 | 4 |

**A5** **I feel content**

| Not at all | Somewhat | Moderately | Very much |
|---|---|---|---|
| 1 | 2 | 3 | 4 |

**A6** **I am worried**

| Not at all | Somewhat | Moderately | Very much |
|---|---|---|---|
| 1 | 2 | 3 | 4 |

For peer review only

**SECTION B – YOUR WORRIES ABOUT DEVELOPING BREAST CANCER**
Please read the statements below and circle the number below each statement that best indicates your current level of worry about getting breast cancer someday:

**B1** **How often have you thought about your chances of getting breast cancer?**

| Never | Rarely | Sometimes | Almost all the time |
|---|---|---|---|
| 1 | 2 | 3 | 4 |

**B2** **How often have these thoughts affected your mood?**

| Never | Rarely | Sometimes | Almost all the time |
|---|---|---|---|
| 1 | 2 | 3 | 4 |

**B3** **How often have these thoughts interfered with your ability to do daily activities?**

| Never | Rarely | Sometimes | Almost all the time |
|---|---|---|---|
| 1 | 2 | 3 | 4 |

**B4** **How concerned are you about the possibility of getting breast cancer one day?**

| Not at all | A little | Somewhat | A lot |
|---|---|---|---|
| 1 | 2 | 3 | 4 |

**B5** **How often do you worry about developing breast cancer?**

| Never | Rarely | Sometimes | Almost all the time |
|---|---|---|---|
| 1 | 2 | 3 | 4 |

**How much of a problem is this worry?**

**B6**

| Not at all | A little | Somewhat | A lot |
|---|---|---|---|
| 1 | 2 | 3 | 4 |

For peer review only

**SECTION C – YOUR PERCEPTION OF BREAST CANCER RISK**
Please tick **ONE** of the statements below that best describes your breast cancer risk in relation to other women of a similar age:

**C1** **Compared to other women my age, I believe my risk of developing breast cancer in the next 10 years is…**

- ❑ Much higher
- ❑ A bit higher
- ❑ About the same
- ❑ A bit lower
- ❑ Much lower

**SECTION D – YOUR ATTITUDES TOWARD BREAST CANCER RISK ASSESSMENT**
Please read the statement and items below and circle the number that best indicates how you feel about participating in breast cancer risk assessment right now, at this moment:

**D1** **Taking part in breast cancer risk assessment was…**

| | | | | |
|---|---|---|---|---|
| Entirely good | Mainly good | Neither good nor bad | Mainly bad | Entirely bad |
| 1 | 2 | 3 | 4 | 5 |
| Entirely beneficial | Mainly beneficial | Neither beneficial nor harmful | Mainly harmful | Entirely harmful |
| 1 | 2 | 3 | 4 | 5 |
| Entirely important | Mainly important | Neither important nor unimportant | Mainly unimportant | Entirely unimportant |
| 1 | 2 | 3 | 4 | 5 |

For peer review only

**SECTION E – YOUR SATISFACTION WITH DECISION TO PARTICIPATE IN BREAST CANCER RISK ASSESSMENT**
Please read the statement below and then circle the most appropriate number below the statement to indicate how satisfied you are with your decision to participate in breast cancer risk assessment.

**E1** **The decision to participate in breast cancer risk assessment was a good decision for me**

| Strongly agree | Agree | Neither agree nor disagree | Disagree | Strongly disagree |
|---|---|---|---|---|
| 1 | 2 | 3 | 4 | 5 |

***Thank you for completing this questionnaire.***
***Please return your completed questionnaire to the study team in the pre-paid envelope provided.***

**Sources of information and support**

You may find some of the following sources of information and support useful if you have any concerns about breast health.

**CoppaFeel!**

Website: https://coppafeel.org/

They have a section that provides guidance on checking your breasts: https://self-checkout.coppafeel.org/onboarding

**Breast Cancer Now**

Website: https://breastcancernow.org/

They have a section where you can ask any questions you have relating to breast health:

https://forum.breastcancernow.org/t5/Ask-Our-Nurses/ct-p/Asknurses

They also offer a free, confidential helpline to answer questions about breast cancer or breast health – 0808 800 6000 (Text relay prefix – 18001)

For peer review only

**Supplementary file 4.** Interview topic guide

**Opening questions**

As you know, we are interested in what women think about the offer of finding out their breast cancer risk as part of the BCAN-RAY study. To start, can you tell me anything about whether breast cancer risk is something you have thought about before being invited to join the BCAN-RAY study?

I understand you were invited to have your breast cancer risk assessed; can we go back to that point and tell me what that was like? What did you think at that point?

How did you make the decision to take part in breast cancer risk assessment?
Prompts:

- Were there are aspects of the BCAN-RAY study that made you question whether to take part (any concerns)?
- Can you tell me anything about why you wanted to know your risk? Anything personal to you?
- How did you receive the invite (as a letter from GP practice if no recall)? What do you think about receiving it that way? How do you think that influenced your decision to have your breast cancer risk assessed?
- (if not already come up) When you were deciding, did you discuss it with anyone (friend / family / study team / GP)?
- Did you feel you had all the information you needed to make a decision about whether to take part? If not, what would have been helpful to know?

**Questions relating to risk assessment process**

Can you tell me what you had to do once you joined the study? Could you tell me about what happened when you had your breast cancer risk assessed?
Probes: What was it like / can you tell me anything about it
Prompts:

- Completing the risk factors questionnaire e.g. how easy was it to access, can you remember what it was asking you to do, were any questions unclear, ability to answer the questions more generally, did you find any questions uncomfortable to answer, did you get any support to help with this part of the study
- What happened once you completed the questionnaire? What was that time-period like?
- Attending the appointment at the hospital (spit sample, mammogram) e.g. what did you think about how the appointment was arranged
- Waiting for the risk feedback results (up to 16 weeks turnaround) e.g. how were you feeling during this time, what did you think about the length of time you had to wait, did you look for any information related to breast cancer during this time
- Receiving the risk feedback (a letter if no recall)
- Contents/wording of the letter (thoughts, feelings and understanding) e.g. did the feedback you received match your expectations in terms of what you thought you would be told
- Logging back into the app to view detailed risk feedback (if not, why not)

- Personal meaning of risk category received e.g. what do you remember about your risk result, how would you describe the risk, how do you feel about the factors that contributed to your risk (increased risk), how did it make you feel, was it something you expected, how do you feel about your risk today/now
- Discussing risk feedback with others (friends / family / healthcare professionals)
  - Did you talk about your risk feedback with anyone in the study team / outside the study team? If yes/no, why? What did you discuss?
  - What did you think of the support provided at this point?
- (increased risk) Experience of risk consultation
  - What did you think about the option to receive an appointment to discuss your risk if it was increased?

After you received your risk feedback, did you do anything differently that you thought might reduce your breast cancer risk?
Prompts:

- (all) Health behaviours
- (increased risk) Recommendation to contact medical doctors to discuss risk reducing medication / additional screening
- (increased risk) Deciding whether to have risk reducing medication
- (increased risk) Deciding whether to have additional screening

Looking back, was there anything that caused any concerns during the risk assessment process? Is there anything you would have preferred to happen in a different way?

Looking back, how do you feel about having made the decision to take part in breast cancer risk assessment?
Prompts:

- Did you understand what was involved when you made the decision to participate?
  Probe: did you have sufficient information?

The way breast cancer risk is calculated changes over time as we learn more about new risk factors. As we are trying to find out whether using a low dose mammogram helps to identify younger women at risk of developing breast cancer, towards the end of the study you will receive updated risk feedback. At this point, your risk might change. What are your thoughts about this? Why?

We are trying to figure out whether introducing a breast cancer risk assessment service for women aged 30 to 39 years is a good or bad idea. What are your thoughts about this? Why? Would you recommend a breast cancer risk assessment service to friends and family members of a similar age?

**<u>Finishing comments</u>**

Thanks for your time today. We do really appreciate it.

- Is there anything else you want to add?

For peer review only

- Is there anything you thought you would talk about today which you haven't had a chance to say and want to mention?
- Do you have any questions for me?

Thanks again. The interview will be typed up by a partner transcription company we use. When this is done, we will remove anything you have said that could identify you such as names or places and you will be given a fake name. If you have any questions feel free to contact the research team at any time *[point out contact details]*.

For peer review only

**Supplementary file 2.** Detailed description of self-reported measures of potential harms and benefits of participation in breast cancer risk assessment

| **Measures** | **Description** |
|---|---|
| State anxiety (36) and cancer worry (37) | To determine whether increased distress is a harm of participating in breast cancer risk assessment, we will compare levels of general anxiety and breast cancer worry between average and increased risk women and across time to evaluate short as well as longer term effects. One might expect changes in distress, particularly amongst women being identified as increased risk, as the result may be unexpected because of a lack of family history of the disease. General state anxiety will be assessed using the six-item short-form of the state scale of the State Trait Anxiety Inventory (STAI) (36), with participants responding to six statements (e.g. "I feel tense") about how they currently feel by selecting one of the following response options "not at all", "somewhat", "moderately" and "very much".<br><br>Breast cancer worry will be assessed using the Lerman Cancer Worry Scale (37). The scale consists of six statements such as: "how often do you worry about developing breast cancer?". Participants will |

For peer review only

| | |
|---|---|
| | endorse one of the following response options for items 1-3 and 5: “never”, “rarely”, “sometimes”, and “almost all the time”. For items 4 and 6, participants select one option from “not at all”, “a little”, “somewhat”, and “a lot”. Both scales have previously been used in similar studies evaluating the psychological impact of receiving breast cancer risk estimates (26, 27). |
| Risk perception (38) | Perceived comparative risk of developing breast cancer will be assessed using a single item whereby women will be asked to rate their risk of developing breast cancer in the next 10 years, compared with other women of their age (38). Participants will select one of the following response options: “much higher”, “a bit higher”, “about the same”, “a bit lower”, and “much lower”. |
| Attitudes towards breast cancer risk assessment (39) | Attitudes towards breast cancer risk assessment will be assessed following a standard approach (39). Three items will be used to assess affective (feelings towards the behaviour) and instrumental (evaluation of the behaviour’s outcomes) attitudes. Women will be asked to indicate the extent to which they view risk assessment as good/beneficial/important, with response options including: “entirely good”, “mainly good”, “neither good nor bad”, “mainly bad”, and “entirely bad”. |

For peer review only

| | |
|---|---|
| Knowledge | No validated measure has been developed for the assessment of breast cancer risk assessment knowledge. Therefore, we decided to create a measure focusing on knowledge of the breast cancer risk assessment process to assess the potential benefit of increased knowledge and inform future implementation. The measure is informed by data on potential misunderstandings of the breast cancer risk assessment process identified from a content analysis of qualitative data collected in the context of optimising the delivery of breast cancer risk assessment in the BCAN-RAY study (28). The measure consists of three questions that map onto the potential misunderstandings identified, namely eligibility for risk assessment, the purpose of the mammogram and access to screening and preventive strategies. Subjective knowledge will be assessed with a single item that asks women to rate how informed they feel about their breast cancer risk, from “very well informed”, “quite well informed”, “quite uninformed”, and “not very informed at all”. |
| Satisfaction with risk feedback information (40) | Satisfaction with risk feedback information will be assessed using four items from a published scale (40) that has been used previously in breast cancer risk-stratification research (26, 27). Women will be asked how well informed they feel about their breast cancer risk, how satisfied they are with the amount of information given, how confusing they found it, and how clear they found the |

For peer review only

| | |
|---|---|
| | information. Participants will select one of the following response options for each item: “strongly agree”, “agree”, “agree somewhat”, “undecided”, “somewhat disagree”, “disagree”, and “strongly disagree. |
| Satisfaction with decision to participate in breast cancer risk assessment (41) | Participants’ remorse or distress over their decision to take part in breast cancer risk assessment will be assessed using a single item adapted from the Decision Regret Scale (41): “The decision to participate in breast cancer risk assessment was a good decision for me”. Response options will be “strongly agree”, “agree”, “neither agree nor disagree”, “disagree”, and “strongly disagree”. |

For peer review only

BMJ Open

# The feasibility and acceptability of offering breast cancer risk assessment to general population women aged 30-39 years: A mixed-methods study protocol

| | |
|---|---|
| Journal: | *BMJ Open* |
| Manuscript ID | bmjopen-2023-078555.R1 |
| Article Type: | Protocol |
| Date Submitted by the Author: | 24-Nov-2023 |
| Complete List of Authors: | Hindmarch, Sarah; The University of Manchester, Manchester Centre for Health Psychology, Division of Psychology and Mental Health, School of Health Sciences, Faculty of Biology, Medicine and Health<br>Howell, Sacha; The University of Manchester, Division of Cancer Sciences, Faculty of Biology, Medicine and Health, University of Manchester, Manchester Academic Health Science Centre<br>Usher-Smith, Juliet; University of Cambridge, Primary Care Unit, Department of Public Health and Primary Care<br>Gorman, Louise; The University of Manchester, NIHR Greater Manchester Patient Safety Research Collaboration, Division of Population Health, Health Services Research & Primary Care, Faculty of Biology, Medicine and Health<br>Evans, D. Gareth ; The University of Manchester, Manchester Academic Health Science Centre, Division of Evolution and Genomic Sciences, School of Biological Sciences, Faculty of Biology, Medicine and Health<br>French, David; The University of Manchester, Manchester Centre for Health Psychology, Division of Psychology and Mental Health, School of Health Sciences, Faculty of Biology, Medicine and Health |
| <b>Primary Subject Heading</b>: | Oncology |
| Secondary Subject Heading: | Public health, Qualitative research, Health services research |
| Keywords: | Breast tumours < ONCOLOGY, Feasibility Studies, QUALITATIVE RESEARCH, Risk Factors, Surveys and Questionnaires, Health Equity |
| | |

SCHOLARONE™
Manuscripts

# The feasibility and acceptability of offering breast cancer risk assessment to general population women aged 30-39 years: A mixed-methods study protocol

Sarah Hindmarch[1*]; Sacha J. Howell[2]; Juliet A. Usher-Smith[3]; Louise Gorman[4]; D. Gareth Evans[5,6]; David P. French[1]

[1]Manchester Centre for Health Psychology, Division of Psychology and Mental Health, School of Health Sciences, Faculty of Biology, Medicine and Health, University of Manchester, Manchester, UK

[2]Division of Cancer Sciences, Faculty of Biology, Medicine and Health, University of Manchester, Manchester Academic Health Science Centre, Manchester, UK

[3]Primary Care Unit, Department of Public Health and Primary Care, University of Cambridge, Cambridge, UK

[4]NIHR Greater Manchester Patient Safety Research Collaboration, Division of Population Health, Health Services Research & Primary Care, Faculty of Biology, Medicine and Health, University of Manchester, Manchester, UK

[5]University of Manchester, Manchester Academic Health Science Centre, Division of Evolution and Genomic Sciences, School of Biological Sciences, Faculty of Biology, Medicine and Health, Manchester, UK

[6]St Mary's Hospital, Manchester University NHS Foundation Trust, Manchester Academic Health Science Centre, North West Genomics Laboratory Hub, Manchester Centre for Genomic Medicine, Manchester, UK

Corresponding author, sarah.hindmarch@manchester.ac.uk



For peer review only - http://bmjopen.bmj.com/site/about/guidelines.xhtml

Word count: 4,633

## Abstract

**Introduction:** Breast cancer incidence starts to increase exponentially when women reach 30-39 years, hence before they are eligible for breast cancer screening. The introduction of breast cancer risk assessment for this age group could lead to those at higher risk receiving benefits of earlier screening and preventive strategies. Currently, risk assessment is limited to women with family history of breast cancer only. The BCAN-RAY study is evaluating a comprehensive breast cancer risk assessment strategy for women aged 30-39 years incorporating a questionnaire of breast cancer risk factors, low-dose mammography to assess breast density, and polygenic risk. The present study will assess the feasibility and acceptability of the BCAN-RAY risk assessment strategy.

**Methods and analysis:** The present study involves women undergoing risk assessment as part of the BCAN-RAY case-control study ($n$ = 750). They will be aged 30-39 years without a strong family history of breast cancer and invited to participate via general practice. A comparison of uptake rates by socioeconomic status and ethnicity between women who participate in the BCAN-RAY study and women who decline participation will be conducted. All participants will be asked to complete self-report questionnaires to assess key potential harms including increased state anxiety (STAI), cancer worry (Lerman Cancer Worry Scale), and satisfaction with decision to participate (Decision Regret Scale), alongside potential benefits such as feeling more informed about breast cancer risk. A sub-sample of approximately 24 women (12 at average risk and 12 at increased risk) will additionally participate in semi-structured interviews to understand the acceptability of the risk assessment strategy and identify any changes needed to it to increase uptake.



For peer review only - http://bmjopen.bmj.com/site/about/guidelines.xhtml

**Ethics and dissemination:** Ethical approval was granted by North West - Greater Manchester West Research Ethics Committee (reference: 22/NW/0268). Study results will be disseminated through peer-reviewed journals, conference presentations and charitable organisations.

**Trial registration:** NCT05305963.

**Keywords:** risk assessment, breast cancer, psychological impact, health inequalities, acceptability

## Article Summary

### Strengths and limitations of this study

- This is the first study to examine the feasibility and acceptability of comprehensive breast cancer risk assessment for general population women aged 30-39 years.
- This study uses a mixed methods design; the combination of qualitative and quantitative data will result in a more comprehensive understanding of the processes affecting implementation.
- Outcome measures assessing potential harms and benefits of participating in breast cancer risk assessment will be collected at three timepoints, allowing for assessment of short and long term effects.
- The quality and completeness of ethnicity data across general practices may be suboptimal for the planned analyses.
- As this is a feasibility study, no information about the effectiveness of breast cancer risk assessment will be provided.



For peer review only - http://bmjopen.bmj.com/site/about/guidelines.xhtml

## Introduction

Breast cancer is the most common cancer diagnosed worldwide for women, with increasing incidence rates observed in pre-menopausal women in recent years (1, 2). This is concerning as breast cancer is more frequently lethal in younger women than in those diagnosed aged over 50 years (10-year survival aged <40 years at diagnosis 70% vs 87% in those >50 years) (3). This is due to a combination of factors, notably later stage at presentation and a greater proportion of women developing more aggressive breast cancer subtypes (4-6). Breast cancer is the leading cause of death in women aged 35-50 years in the UK (7). Therefore, there is a pressing need to identify younger women at increased risk of developing breast cancer so they can be offered screening and preventive strategies (8).

Assessment of an individual's breast cancer risk is one proposed approach for identifying young women eligible for screening and preventive strategies (9). In the UK, a strong family history of breast cancer or known high risk genetic variant in a close relative is the only criteria by which women aged under 50 years can access screening and preventive strategies prior to a diagnosis of breast cancer (10). However, at least 65% of women who develop breast cancer before the age of 50 years do not have such a family history and are not currently identified as being at increased risk (3, 11).

The reliance on family history belies the progress over recent decades in the identification of additional breast cancer risk factors including those related to reproductive and hormonal history, alcohol consumption, polygenic risk scores and mammographic density. These additional factors have been incorporated into risk prediction models, resulting in improved discrimination across all age groups (12-15). In the UK, the PROCAS study confirmed it was possible to accurately estimate a woman's individual risk of developing breast cancer at the


For peer review only - http://bmjopen.bmj.com/site/about/guidelines.xhtml

time of mammographic screening using a self-reported questionnaire of breast cancer risk factors and assessment of mammographic density and polygenic risk (16). Using this comprehensive approach to risk assessment identified 18% of women as being at least moderate risk of developing breast cancer in comparison to only 3.7% using family history alone (17). Therefore, a greater number of women were identified who would be eligible for consideration of screening and preventive strategies (10). Trials are underway internationally to establish the potential effectiveness of risk-based screening strategies for women attending breast cancer screening programmes over the age of 40 years (18, 19). However, inclusion of breast cancer risk assessment at the time of national mammographic screening programmes will miss younger women eligible for screening and preventive strategies. Therefore, the introduction of comprehensive breast cancer risk assessment from an earlier age is currently being considered.

A recent review determined that breast cancer risk assessment for women under 50 years currently satisfies many of the key principles for screening (20). However, uncertainties remain with respect to the optimal strategy for implementation and potential impact of the invitation process on health inequalities. The Breast CANcer Risk Assessment in Younger women (BCAN-RAY) case-control study (NCT05305963) aims to evaluate a comprehensive breast cancer risk assessment strategy amongst a diverse ethnic and socioeconomic population of women aged 30-39 years without a strong family history of breast cancer (21). The BCAN-RAY study aims to primarily assess the impact of mammographic density on breast cancer risk in this age group. To address this, we have developed a low-dose mammogram technique which uses a tenth or less of the radiation dose of a full dose screening mammogram making it safer. Furthermore, an automated method of analysis not



For peer review only - http://bmjopen.bmj.com/site/about/guidelines.xhtml

requiring radiologist review will be utilised, removing the risk of unnecessary recall for additional imaging. This approach has been shown to be accurate in younger women (22).

The risk assessment strategy thereby consists of a questionnaire of breast cancer risk factors, low-dose mammography to measure mammographic density, and a saliva sample to assess polygenic risk and the presence of pathogenic variants in high and moderate-risk genes. The breast cancer risk assessment strategy adopted in the BCAN-RAY study is herein referred to as the BCAN-RAY approach. Women with a strong family history of breast cancer are ineligible to participate because they can access screening and preventive strategies through referral to Family History, Risk and Prevention Clinics (FHRPCs). Women identified as being at increased risk will be offered an appointment at a FHRPC to discuss their risk result further and potential management options. Options in the UK include access to breast screening from the age of 40 years (if 10-year risk reaches 3% by 40) and preventive strategies such as weight loss or weight gain prevention interventions and risk-reducing medication. Uptake of these screening and preventive strategies by younger women has the potential to facilitate earlier detection of breast cancer and reduce breast cancer mortality (9).

In line with the MRC Framework for Developing and Evaluating Complex Interventions (23), it is imperative to assess the feasibility of the BCAN-RAY approach in order to inform future decisions about implementation. One key consideration is a need to assess whether the invitation process exacerbates health inequalities through lower recruitment of ethnic minority populations and women from low socioeconomic backgrounds. Previous efforts to implement risk assessment at the time of mammographic screening have demonstrated

For peer review only



For peer review only - http://bmjopen.bmj.com/site/about/guidelines.xhtml

these problems (24). This is important to consider as addressing ethnic disparities in breast cancer mortality has been recognised as a key research priority (25).

Secondly, potential harms and benefits need to be identified. There is now considerable evidence on the effects of providing breast cancer risk estimates to women aged 47-73 years recruited via the NHS Breast Screening Programme. These data indicate that women subsequently had more accurate perceptions of risk with no evidence of significant adverse effects on anxiety or cancer worry (26, 27). Nevertheless, there is a need to show an absence of adverse effects when setting up a new programme with younger women for several reasons. First, one might expect more acute distress amongst younger women at increased risk as the result may be more unexpected because of a lack of family history of the disease, suggesting anxiety and cancer worry are important outcomes to assess. Second, due to the potential implications of being identified as at increased risk for younger women in terms of reproductive decision-making, a possible harm could be that participants experience remorse or distress over their decision to take part in breast cancer risk assessment. In terms of benefits, it is anticipated that women will feel more informed about breast cancer risk as a result of participation which will enable them to make informed choices about subsequent risk management options.

Finally, it is important to consider acceptability of the BCAN-RAY approach to women aged 30-39 years to optimise the likelihood of future implementation being successful. If the processes of invitation, risk assessment and feedback are unacceptable, then the potential benefits will not be realised. For this study, acceptability is defined as the extent to which women receiving breast cancer risk assessment consider it to be appropriate, based on

For peer review only



For peer review only - http://bmjopen.bmj.com/site/about/guidelines.xhtml

experienced cognitive and emotional responses to participating in risk assessment, in line with an evidence-based framework of acceptability (28).

We have previously conducted a qualitative study with women aged 30-39 years which suggested that undergoing breast cancer risk assessment was acceptable in principle (29). However, risk assessment was presented as a hypothetical prospect in that study so how women may respond once they have experienced it and any changes required to increase engagement and uptake remain unknown.

The present study aims to examine the feasibility and acceptability of a strategy to offer breast cancer risk assessment to women aged 30-39 years in a diverse ethnic and socioeconomic geographical region. A mixed-methods approach will be adopted in order to capitalise on the strengths of both quantitative and qualitative methods, resulting in a more comprehensive understanding of the processes affecting implementation (30). Specific objectives of this study are to:

a) Examine uptake rates according to socioeconomic status and ethnicity to determine impact of the invitation process on health inequalities
b) Identify potential harms and benefits of participation in breast cancer risk assessment
c) Understand the acceptability of the BCAN-RAY approach

## Methods

### Design

BCAN-RAY is a case-control study (21). Approximately one thousand women will be recruited between May 2023 and May 2025, 250 women diagnosed with breast cancer



For peer review only - http://bmjopen.bmj.com/site/about/guidelines.xhtml

when they were aged 30-39 years (cases) and 750 controls currently aged 30-39 years without a strong family history of breast cancer. The present feasibility study involves the control participants only and uses three different analyses to address the three objectives.

***a. Health inequalities assessment***

A between-subjects comparison will be made between women who participate in the BCAN-RAY study and women who decline participation according to socioeconomic status and ethnicity.

***b. Identification of potential harms and benefits***

Quantitative questionnaires will be administered to each woman at three timepoints; baseline, 6 weeks post risk feedback and 6 months post risk feedback. A between-subjects comparison will be made between average and increased risk women for outcomes assessed at multiple timepoints.

***c. Understanding acceptability***

A cross-sectional qualitative design will be adopted employing one-to-one semi-structured interviews.

**Setting and participants**

All general practices across Greater Manchester have been approached for participation in BCAN-RAY as participant identification centres. An electronic database search will be conducted by each practice to identify women aged 30-39 years predicted to meet eligibility criteria. All potentially eligible women will be invited. We expect to recruit a diverse sample in terms of ethnicity and socioeconomic status given that Greater Manchester has one of



For peer review only - http://bmjopen.bmj.com/site/about/guidelines.xhtml

the most ethnically diverse populations in the UK in addition to some of the most deprived areas (31, 32). Furthermore, general practices in areas of higher ethnic and socioeconomic diversity will be prioritised during setup. Participants meet BCAN-RAY study inclusion criteria if they are (1) born biologically female, (2) aged 30-39 years, and (3) able to provide informed consent. Participants cannot take part if they meet any of the exclusion criteria outlined in Table 1. A series of eligibility checks will be conducted which are described in the next section.

**Table 1.** Study exclusion criteria

| |
|---|
| Strong family history of breast cancer, defined as a first degree relative diagnosed with breast cancer under the age of 50 or two or more second-degree relatives diagnosed with breast cancer at any age |
| Already under follow up in a breast cancer family history clinic or have a known mutation in a moderate or high-risk breast cancer gene |
| Any prior malignancy (excluding non-melanoma skin cancer) |
| Had a double mastectomy (both breasts removed) |
| Breast implants or breast augmentation surgery |
| Currently pregnant |
| Currently breast-feeding or stopped breast-feeding less than six months ago |
| Any condition that would make breast cancer risk assessment inappropriate such as a severe psychiatric or physical illness (assessed by the individual responsible for identifying and inviting women) |
| Unable to understand written English |

## Procedure



For peer review only - http://bmjopen.bmj.com/site/about/guidelines.xhtml

***BCAN-RAY study***

Participating general practices will send postal invitations to eligible women. The BCAN-RAY invitation letter will contain a QR code and web-link to access the participant information sheet and instructions directing prospective participants to the risk assessment web-based application. Once participants have consented to the study online, they will be directed to the BCAN-RAY risk factors questionnaire based on the Tyrer-Cuzick algorithm (33). Participants will be able to answer part of the questionnaire, save and return to it at a later date. If a participant does not have access to the internet or is having difficulty completing the questionnaire, they can provide their answers via telephone to the study team who will manually input the participants' responses into the web-based application. If a strong family history of breast cancer (as defined in Table 1) is identified during completion of the risk factors questionnaire, participants will be referred back to their GP for FHRPC referral and their participation in the BCAN-RAY study will end. Following submission of consent and the risk factors questionnaire, participants will be contacted by telephone or email to arrange the risk assessment appointment which will take place at the Nightingale Centre, part of the Manchester University NHS Foundation Trust. Before an appointment is offered, eligibility to take part will be checked by a member of the study team using an eligibility checklist based on self-report. Women who meet any of the exclusion criteria will be withdrawn from the study. Before the appointment, participants will be sent a saliva sample collection tube in the post and asked to bring the saliva sample along to the appointment, which will be analysed for polygenic risk score (SNP313) and the presence of pathogenic variants in high and moderate-risk genes. At the appointment, a final eligibility check will be conducted based on self-report in case any of the information provided in the risk factors questionnaire



For peer review only - http://bmjopen.bmj.com/site/about/guidelines.xhtml

has changed since the participant completed it. Once eligibility has been confirmed, participants will undergo low-dose mammography (two views of one breast only). Breast density will be calculated using a new technique called predicted visual assessment score (pVAS). pVAS is an automated method of assessing mammograms using artificial intelligence techniques (22, 34). A risk feedback letter will be generated based on the answers participants give in their questionnaire, the results of genetic testing and mammographic density. The risk feedback letter will inform women that they are at “average” risk (< 3% 10-year risk) or “increased” risk (≥ 3% 10-year risk). The decision to not provide women with information about the relative impact of each risk component in the risk feedback letters was informed by findings of a qualitative study we conducted with women who matched the intended recipients of the feasibility study (29). This study investigated information and support needs with respect to breast cancer risk assessment and risk communication and found that information about the factors contributing to risk was perceived as interesting but generally unhelpful when receiving initial notification of the risk result. Instead, information about what would happen next in terms of proactive risk management was considered most important. Each letter therefore focuses on explaining the implications of the risk result (see supplementary file 1). Participants identified as at increased risk will be offered an appointment at a FHRPC to discuss their risk result further with a breast clinician with expertise in risk assessment, screening and prevention. At this appointment, potential management options including earlier access to breast screening and risk-reducing medication will be discussed. All participants will receive their risk feedback letter within 16 weeks of the risk assessment appointment, along with leaflets providing additional detail on ways of reducing breast cancer risk, signs and symptoms of breast cancer and breast awareness. An updated risk feedback letter will be sent at the end of the study once the



For peer review only - http://bmjopen.bmj.com/site/about/guidelines.xhtml

magnitude of risk associated with density is determined more accurately in this age group using all case control subjects. The timeline from the participant perspective is shown in Figure 1.

INSERT FIGURE 1: Timeline of feasibility study integrated with BCAN-RAY

***a. Health inequalities assessment***

GPs from participating general practices will extract self-reported ethnicity (where available) and deprivation information based on residential postcode for all women invited to take part in the BCAN-RAY study so that these characteristics can be compared between those who participate in the study and those who decline participation. They will provide this aggregated, non-identifiable data to the research team. No personally identifiable data will be shared with the research team as we predict the majority of women invited will not consent to the study. A member of the research team will then extract the same information from the BCAN-RAY study database for all participants.

***b. Identification of potential harms and benefits***

Once participants have submitted the risk factors questionnaire on the web-based application, they will be directed to complete the baseline harms and benefits questionnaire on Qualtrics (https://www.qualtrics.com/uk/). If the baseline questionnaire has not been completed by the time a member of the study team rings the participant to arrange their risk assessment appointment, a reminder to do so will be enclosed with their appointment confirmation letter. Any remaining non-completers will be asked to complete the questionnaire online or via paper in the waiting room before their risk assessment appointment.



For peer review only - http://bmjopen.bmj.com/site/about/guidelines.xhtml

The same women will be asked to complete follow up questionnaires 6 weeks and 6 months after they have received their risk feedback. Women will be asked to input their unique BCAN-RAY study ID and their date of birth at the beginning of each questionnaire to ensure responses can be linked. Participants are able to request paper copies of the follow up questionnaires to be sent to them via post if preferred. The data recorded on paper copies of all questionnaires will be manually inputted into the Qualtrics platform by a member of the study team. If the follow up questionnaires have not been completed by two weeks after the initial invitations, a reminder to complete the questionnaire will be sent via email or letter.

***c. Understanding acceptability***

A purposive sample of average and increased risk women who complete the baseline questionnaire and have agreed to be contacted will be sent an invitation to participate in a semi-structured interview. Demographic characteristics and responses to questionnaires will guide sampling to allow variation in ethnicity, socioeconomic status, and anxiety levels of participants. Average risk women will be invited for interview 1 month after receiving their risk feedback letter. Increased risk women will be invited for interview 3 months after receiving their risk feedback letter. This gives women at increased risk the chance to explore extra screening options or medications prior to the interview and minimises any influence participating in the interview may have on decision-making. We will aim to recruit up to 24 women to these interviews (up to 12 women in each risk category). If no response is received following the initial invitation, a second invitation will be sent approximately 3-4 weeks later.



For peer review only - http://bmjopen.bmj.com/site/about/guidelines.xhtml

Interviews will last approximately 40-60 minutes and will be conducted face-to-face or over the telephone according to each participant's preference. For face-to-face interviews, written consent will be obtained. For telephone interviews, verbal consent will be obtained over the telephone before the interview begins and recorded in a separate audio file. Interviews will be audio recorded and transcribed verbatim using an accredited transcription company. Participants will be compensated for their time with a £20 shopping voucher.

## Measures

### *a. Health inequalities assessment*

Residential postcode, a proxy measure of socioeconomic status, will be converted into deprivation deciles using the Index of Multiple Deprivation (IMD), a measure of relative deprivation for small areas in England (35). Where available, ethnicity data will be mapped onto the five high-level ethnic categories used in the 2021 Census for England (White, Asian/Asian British, Black/African/Caribbean/Black British, Mixed/Multiple, and Other ethnic group), in line with the current ethnicity harmonised standard (36). Missing data will be captured under two additional categories of refusal to provide information about ethnic group and no data available.

### *b. Identification of potential harms and benefits*



For peer review only - http://bmjopen.bmj.com/site/about/guidelines.xhtml

The self-reported measures of potential harms and benefits of participation in breast cancer risk assessment to be completed by participants are shown in Table 2. A detailed description of each of these measures is provided in supplementary file 2. Supplementary file 3 contains a copy of each questionnaire.

**Table 2.** Self-reported measures to be assessed, at each of the three timepoints

| Baseline | 6 weeks post risk feedback | 6 months post risk feedback |
|---|---|---|
| State anxiety (37) | State anxiety (37) | State anxiety (37) |
| Cancer worry (38) | Cancer worry (38) | Cancer worry (38) |
| Risk perception (39) | Risk perception (39) | Risk perception (39) |
| Attitudes towards risk assessment (40) | | Attitudes towards risk assessment (40) |
| | Knowledge[a] | |
| | Satisfaction with risk feedback information (41) | |
| | | Satisfaction with decision to participate in breast cancer risk assessment (42) |

[a]Assessed by a measure the research team has created as no validated measure available (see supplementary file 2 for more information about development of this measure)

### c. Understanding acceptability



For peer review only - http://bmjopen.bmj.com/site/about/guidelines.xhtml

Topic guide development was informed by the aims of the study and a review of the literature. An initial draft was developed by the lead author, a doctoral student in health psychology with qualitative health services research experience. Feedback on this draft was obtained from public contributors and members of the research team (DPF and JAU-S) who have research expertise in breast cancer and screening services, primary care and health services research, health psychology, and qualitative methods. The content and structure of the topic guide was revised in line with the feedback received. Participants will be asked about their experience of the risk assessment process including how acceptable they found it, their views on the materials developed for BCAN-RAY, and how the risk assessment process could be improved in terms of delivery/access and provision of information and support (see supplementary file 4). Furthermore, women will be asked to discuss any actions they have considered and/or made as a result of participating in BCAN-RAY (e.g. lifestyle modifications, additional screening and risk-reducing medication).

**Data analysis**

***a. Health inequalities assessment***

The Chi-squared test will be used to compare uptake rates by ethnicity and socioeconomic status (assessed by IMD deciles) between women who participate in the BCAN-RAY study and women who decline participation. To ensure sufficient instances in each group, IMD deciles will be collapsed into quintiles and ethnicity will be collapsed into 6 subgroups (White, Asian, Black, Mixed or Multiple, Other and Missing).

***b. Identification of potential harms and benefits***

For peer review only



For peer review only - http://bmjopen.bmj.com/site/about/guidelines.xhtml

The main analyses will focus on comparing the responses of the two groups of women provided with different risk estimates (average and increased) for outcomes assessed at multiple timepoints (i.e. anxiety, cancer worry, risk perceptions and attitudes towards breast cancer risk assessment). ANCOVA will be used, with baseline responses to the same variables, age and IMD deciles as covariates. Analyses will be conducted on all questionnaire measures at 6 weeks and 6 months, with the 6-month state anxiety measure being the primary outcome.

Measures administered at only one timepoint (knowledge, satisfaction with information received and satisfaction with decision to participate in breast cancer risk assessment) will be compared between the two groups of women provided with different risk estimates (average or increased). ANCOVA will be used, with age and IMD deciles as covariates.

All statistical tests will be two-sided and use a significance level of 5%. A “completer only” analysis strategy will be employed. If dropout levels are high, the a priori primary outcome (comparison of 6-month outcome scores between average and increased risk groups) will be repeated using a last occasion carried forward approach to missing data as a sensitivity analysis. Statistical analyses will be performed using SPSS.

***c. Understanding acceptability***

NVivo software will be used to organise the data. Data will be analysed using a manifest level approach to reflexive thematic analysis (43, 44). Thematic analysis involves examining qualitative data to produce themes that summarise and interpret patterns of results. Initial coding will be deductive based on the structured questions in the topic guide to address the objective of whether the BCAN-RAY approach is acceptable. Inductive methods will then be



For peer review only

used to capture additional codes and context to ensure important aspects of the data are not missed. A critical realist approach will be adopted, with the researchers accepting that participants' accounts represent their perception of their reality, which is shaped by and embedded within their cultural context and language (45). An experiential orientation to data interpretation will be adopted that seeks to stay close to participants' meanings and capture these in ways that might be recognisable to them. The analysis will be conducted by the lead researcher with input from other members of the research team and public contributors.

## Sample size estimation

### *a. Health inequalities assessment*

The BCAN-RAY feasibility study aims to recruit approximately 750 women. Based on the results of the latest NHS GP Patient Survey in which 13-19% of those invited by post aged 25-44 responded (46), we conservatively expect a response rate of 10%. Therefore, approximately 7,500 invitations will be sent. If the response rate is lower than expected, more invitations will be sent until at least 750 women have been recruited. This approach will also yield at least 6,750 women who decline participation. Given the geographical spread of the general practices who have provisionally agreed to be involved in the study across different boroughs of Greater Manchester, we expect to recruit a socioeconomically diverse sample (see Table 3).

**Table 3.** Percentage of Lower Super Output Areas (LSOAs) in each deprivation decile across the boroughs of Greater Manchester involved in the BCAN-RAY study[a]

| | Location |
|---|---|



For peer review only - http://bmjopen.bmj.com/site/about/guidelines.xhtml

| Deprivation decile[b] | Trafford | Manchester | Salford | Tameside | Rochdale | Stockport |
|---|---|---|---|---|---|---|
| 1-2 (most deprived) | 8.7% | 59.3% | 48.7% | 42.6% | 44.1% | 16.3% |
| 3-4 | 15.9% | 25.8% | 21.4% | 22.7% | 26.1% | 20% |
| 5-6 | 15.2% | 10.7% | 15.3% | 20.6% | 10.4% | 15.3% |
| 7-8 | 25.3% | 3.9% | 7.3% | 12.1% | 15% | 21.6% |
| 9-10 (least deprived) | 34.8% | 0.4% | 7.3% | 2.1% | 4.5% | 26.9% |

[a]Data sourced from an interactive map created by Greater Manchester Poverty Action (31)

[b]Assessed by the Index of Multiple Deprivation 2019 (35)

***b. Identification of potential harms and benefits***

The sample size for the BCAN-RAY study was based on providing sufficient power to be able to detect an effect of breast density, after adjustment for age and BMI. Therefore, a post hoc analysis was conducted to estimate achieved power with respect to the primary outcome of anxiety at 6 months. Assuming a two-tailed independent samples t-test and follow up questionnaire responses from 400 average risk women and 100 increased risk women, it is estimated that there will be approximately 76% power to detect a small, standardised difference of d = 0.3.

***c. Understanding acceptability***

The sample size for the BCAN-RAY study will provide more than sufficient numbers from which to recruit participants for the acceptability assessment. Whilst we anticipate including



For peer review only - http://bmjopen.bmj.com/site/about/guidelines.xhtml

up to 24 participants in this component of the study (12 at average risk and 12 at increased risk), the decision to stop recruitment will be guided by the concept of ‘information power’. The research team will reflect on the information richness of their dataset throughout data collection to determine when sufficient data has been collected to answer the research question (47).

**Public involvement**

A public involvement group of 11 women aged 30-39 years was established in September 2021 to inform the development of research aimed at identifying young women at increased risk of breast cancer including the BCAN-RAY study. Five women reviewed the study documentation (participant information sheet, consent form, study invite letter, risk feedback letters, baseline and follow up questionnaires, and interview topic guide). The content and structure of documentation was revised in line with the feedback received. Changes included the removal of one question from the knowledge measure as it overlapped considerably with the content of one of the other questions and the addition of breast cancer charity contact information to risk feedback letters. We will continue to involve members of the public involvement group in subsequent stages of the research cycle including analysis of interview data and dissemination.

**Ethics and dissemination**

This study was approved by the North West - Greater Manchester West Research Ethics Committee (reference: 22/NW/0268). The study will be performed in accordance with the Declaration of Helsinki, Good Clinical Practice principles and relevant regulations. All participants in BCAN-RAY complete written consent online. All participants will provide informed consent (written if face-to-face, verbal if over telephone) prior to taking part in an



For peer review only - http://bmjopen.bmj.com/site/about/guidelines.xhtml

For peer review only

interview. Quantitative study data will be tracked via participant study IDs. Identifying information will be removed from the interview transcripts and participants will be assigned pseudonyms.

We will disseminate our findings through peer-reviewed journals, conference presentations and charitable organisations. At the time of consent for both the BCAN-RAY study and an interview, participants will be asked to indicate whether they wish to receive a summary of findings. A written lay summary will be produced and sent to those who opt to receive this.

## Discussion

The present research aims to provide evidence on the feasibility of a strategy to offer breast cancer risk assessment based on family history, phenotypic risk factors, polygenic risk and mammographic density to women aged 30-39 years. It will provide information about uptake rates, potential harms and benefits of participation, and the acceptability of the risk assessment strategy including novel insight into the experience of low-dose mammography amongst a population of women not known to be at increased risk of breast cancer.

One key issue that the present research does not cover relates to whether breast cancer risk assessment in younger women is acceptable to healthcare professionals involved in its delivery, which is recognised as an important component of feasibility (23). We have interviewed and conducted focus groups with primary care professionals to understand their views on involvement in breast cancer risk assessment and management and analysis is ongoing. However, as the optimal strategy for implementation remains unclear, it is not yet known who would be responsible for the delivery of risk assessment. Future research investigating alternative strategies for implementation ought to consider the views of



For peer review only - http://bmjopen.bmj.com/site/about/guidelines.xhtml

healthcare personnel involved in delivery to establish likely effects on the healthcare system when implementing risk assessment.

The study will provide valuable information about whether a primary care co-ordinated invitation process is successful at engaging women from diverse socioeconomic and ethnic backgrounds thereby informing the need to consider and evaluate alternative invitation methods prior to further implementation. Furthermore, findings will provide information about the likely harms and benefits of participation in breast cancer risk assessment and identify modifications needed to the risk assessment strategy to increase engagement and uptake in future implementation studies.

Key feasibility issues for implementing risk-stratified screening into routine breast cancer screening have now been identified. The present study provides an important first step in assessing the feasibility of introducing comprehensive breast cancer risk assessment for younger women to enable those identified as being at increased risk access to screening and preventive strategies in the absence of a family history of breast cancer.

## Declarations

### Author Contributions

The BCAN-RAY study was conceived and designed and is being led by SJH and DGE. Funding for BCAN-RAY was led by SJH and DGE, with input from JAU-S and DPF. The feasibility study and participant documentation were designed by SH, SJH, JAU-S and DPF. SH co-ordinated the involvement of public contributors. The present article was drafted by SH. DPF, SJH, LG, JAU-S and DGE provided feedback on versions of the manuscript. All authors read and approved the final manuscript.



For peer review only - http://bmjopen.bmj.com/site/about/guidelines.xhtml

**Competing interests**

The authors declare that they have no competing interests.

**Funding**

This study is sponsored by Manchester University NHS Foundation Trust and funded by grants from Cancer Research UK Alliance for Cancer Early Detection (ref: EDDAMC-2021\100003) and The Christie Charity. The web-based application was developed through charitable donations from the Shine Bright Foundation and Tony Thornley. The low-dose mammogram was developed with a grant from the Medical Research Council's Confidence in Concept funding scheme (2018/19). SH is funded by a Manchester Cancer Research Centre PhD studentship. DPF, DGE and SJH are supported by the NIHR Manchester Biomedical Research Centre (IS-BRC-1215-20007 and NIHR203308). JAU-S is funded by an Advanced Fellowship from the National Institute for Health and Social Care Research (NIHR300861). The views expressed are those of the authors and not necessarily those of the NHS, the NIHR or the Department of Health and Social Care. These funding sources had no role in the design of this study and will not have any role during its execution, analyses, interpretation of the data, or decision to submit results.

**Acknowledgements**

We would like to thank Stephanie Archer who helped with the developmental work and Brian McMillan for advising on ethnicity data collection and reporting in primary care. We also gratefully acknowledge the contributions of our public involvement group.



For peer review only - http://bmjopen.bmj.com/site/about/guidelines.xhtml

# References

1. Heer E, Harper A, Escandor N, Sung H, McCormack V, Fidler-Benaoudia MM. Global burden and trends in premenopausal and postmenopausal breast cancer: a population-based study. Lancet Glob Health. 2020;8(8):e1027-37.

2. Sung H, Ferlay J, Siegel RL, Laversanne M, Soerjomataram I, Jemal A, et al. Global cancer statistics 2020: GLOBOCAN estimates of incidence and mortality worldwide for 36 cancers in 185 countries. CA Cancer J Clin. 2021;71(3):209-49.

3. Copson ER, Maishman TC, Tapper WJ, Cutress RI, Greville-Heygate S, Altman DG, et al. Germline BRCA mutation and outcome in young-onset breast cancer (POSH): a prospective cohort study. Lancet Oncol. 2018;19(2):169-80.

4. Assi HA, Khoury KE, Dbouk H, Khalil LE, Mouhieddine TH, El Saghir NS. Epidemiology and prognosis of breast cancer in young women. J Thorac Dis. 2013;5(Suppl 1):S2-8.

5. Bardia A, Hurvitz S. Targeted therapy for premenopausal women with HR+, HER2− advanced breast cancer: focus on special considerations and latest advances. Clin Cancer Res. 2018;24(21):5206-18.

6. Lian W, Fu F, Lin Y, Lu M, Chen B, Yang P, et al. The impact of young age for prognosis by subtype in women with early breast cancer. Sci Rep. 2017;7(1):11625.

7. Office for National Statistics. Deaths registered in England and Wales: 2021. 2022. www.ons.gov.uk/peoplepopulationandcommunity/birthsdeathsandmarriages/deaths/bulletins/deathsregistrationsummarytables/2021. Accessed 19 Apr 2023.

8. Kudela E, Samec M, Kubatka P, Nachajova M, Laucekova Z, Liskova A, et al. Breast cancer in young women: status quo and advanced disease management by a predictive, preventive, and personalized approach. Cancers. 2019;11(11):1791.

For peer review only

9. Evans D, Brentnall AR, Harvie M, Dawe S, Sergeant JC, Stavrinos P, et al. Breast cancer risk in young women in the National Breast Screening Programme: implications for applying NICE guidelines for additional screening and chemoprevention. Cancer Prev Res. 2014;7(10):993-1001.

10. National Institute for Health and Care Excellence (NICE). Familial breast cancer: classification, care and managing breast cancer and related risks in people with a family history of breast cancer (updated Nov 2019). 2013. https://www.nice.org.uk/guidance/cg164/chapter/Recommendations. Accessed 19 Apr 2023.

11. Eccles BK, Copson ER, Cutress RI, Maishman T, Altman DG, Simmonds P, et al. Family history and outcome of young patients with breast cancer in the UK (POSH study). Br J Surg. 2015;102(8):924-35.

12. Dite GS, MacInnis RJ, Bickerstaffe A, Dowty JG, Allman R, Apicella C, et al. Breast cancer risk prediction using clinical models and 77 independent risk-associated SNPs for women aged under 50 years: Australian breast cancer family registry. Cancer Epidemiol Biomarkers Prev. 2016;25(2):359-65.

13. Evans D, Harkness EF, Brentnall AR, van Veen EM, Astley SM, Byers H, et al. Breast cancer pathology and stage are better predicted by risk stratification models that include mammographic density and common genetic variants. Breast Cancer Res Treat. 2019;176(1):141-8.

14. Hurson AN, Pal Choudhury P, Gao C, Hüsing A, Eriksson M, Shi M, et al. Prospective evaluation of a breast-cancer risk model integrating classical risk factors and polygenic risk in 15 cohorts from six countries. Int J Epidemiol. 2021;50(6):1897-911.

For peer review only

15. Vilmun BM, Vejborg I, Lynge E, Lillholm M, Nielsen M, Nielsen MB, et al. Impact of adding breast density to breast cancer risk models: a systematic review. Eur J Radiol. 2020;127:109019.

16. Evans DGR, Donnelly LS, Harkness EF, Astley SM, Stavrinos P, Dawe S, et al. Breast cancer risk feedback to women in the UK NHS breast screening population. Br J Cancer. 2016;114(9):1045-52.

17. van Veen EM, Brentnall AR, Byers H, Harkness EF, Astley SM, Sampson S, et al. Use of single-nucleotide polymorphisms and mammographic density plus classic risk factors for breast cancer risk prediction. JAMA Oncol. 2018;4(4):476-82.

18. Esserman LJ, Anton-Culver H, Borowsky A, Brain S, Cink T, Crawford B, et al. The WISDOM Study: breaking the deadlock in the breast cancer screening debate. NPJ Breast Cancer. 2017;3(1):34.

19. My Personalized Breast Screening (MyPeBS). ClinicalTrials.gov identifier: NCT03672331. 2018. https://clinicaltrials.gov/ct2/show/NCT03672331. Accessed 19 Apr 2023.

20. Usher-Smith JA, Hindmarch S, French DP, Tischkowitz M, Moorthie S, Walter FM, et al. Proactive breast cancer risk assessment in primary care: a review based on the principles of screening. Br J Cancer. 2023;128:1636–46.

21. Breast CANcer Risk Assessment in Younger women: BCAN-RAY (BCAN-RAY). ClinicalTrials.gov identifier: NCT04336904. 2022. https://clinicaltrials.gov/ct2/show/NCT05305963. Accessed 19 Apr 2023.

22. Squires S, Ionescu G, Harkness E, Mackenzie A, Evans D, Maxwell A, et al., editors. Automatic density prediction in low dose mammography. Proc SPIE 11513, 15$^{th}$



For peer review only - http://bmjopen.bmj.com/site/about/guidelines.xhtml

International Workshop on Breast Imaging; 2020. Available from: https://doi.org/10.1117/12.2564714.

23. Skivington K, Matthews L, Simpson SA, Craig P, Baird J, Blazeby JM, et al. A new framework for developing and evaluating complex interventions: update of Medical Research Council guidance. BMJ. 2021;374:n2061.

24. Evans DGR, McWilliams L, Astley S, Brentnall AR, Cuzick J, Dobrashian R, et al. Quantifying the effects of risk-stratified breast cancer screening when delivered in real time as routine practice versus usual screening: the BC-Predict non-randomised controlled study (NCT04359420). Br J Cancer. 2023;128:2063–71.

25. U.S. Preventive Services Task Force (USPSTF). Breast Cancer: Screening (Draft Recommendation Statement). 2023. https://www.uspreventiveservicestaskforce.org/uspstf/draft-recommendation/breast-cancer-screening-adults#fullrecommendationstart. Accessed 22 May 2023.

26. French DP, McWilliams L, Bowers S, Woof VG, Harrison F, Ruane H, et al. Psychological impact of risk-stratified screening as part of the NHS Breast Screening Programme: multi-site non-randomised comparison of BC-Predict versus usual screening (NCT04359420). Br J Cancer. 2023;128:1548-58.

27. French DP, Southworth J, Howell A, Harvie M, Stavrinos P, Watterson D, et al. Psychological impact of providing women with personalised 10-year breast cancer risk estimates. Br J Cancer. 2018;118(12):1648-57.

28. Sekhon M, Cartwright M, Francis JJ. Acceptability of healthcare interventions: an overview of reviews and development of a theoretical framework. BMC Health Serv Res. 2017;17(1):88.



For peer review only - http://bmjopen.bmj.com/site/about/guidelines.xhtml

29. Hindmarch S, Gorman L, Hawkes RE, Howell SJ, French DP. Optimising the delivery of breast cancer risk assessment for women aged 30–39 years: A qualitative study of women's views. Womens Health. 2023;19.

30. Green CA, Duan N, Gibbons RD, Hoagwood KE, Palinkas LA, Wisdom JP. Approaches to mixed methods dissemination and implementation research: methods, strengths, caveats, and opportunities. Adm Policy Ment. 2015;42(5):508-23.

31. Greater Manchester Poverty Action. Deprivation at a neighbourhood level. 2023. https://www.gmpovertyaction.org/pm2022-imd/. Accessed 22 May 2023.

32. Office for National Statistics. Ethnic group, England and Wales: Census 2021. 2022. https://www.ons.gov.uk/peoplepopulationandcommunity/culturalidentity/ethnicity/bulletins/ethnicgroupenglandandwales/census2021#how-ethnic-composition-varied-across-england-and-wales. Accessed 22 May 2023.

33. Tyrer J, Duffy SW, Cuzick J. A breast cancer prediction model incorporating familial and personal risk factors. Stat Med. 2004;23(7):1111-30.

34. Astley SM, Harkness EF, Sergeant JC, Warwick J, Stavrinos P, Warren R, et al. A comparison of five methods of measuring mammographic density: a case-control study. Breast Cancer Res. 2018;20(1):10.

35. Ministry of Housing, Communities, Local Government. English indices of deprivation 2019. 2019. https://imd-by-postcode.opendatacommunities.org/imd/2019. Accessed 22 May 2023.

36. Government Statistical Service. Ethnicity harmonised standard. 2011. https://analysisfunction.civilservice.gov.uk/policy-store/ethnicity-harmonised-standard/. Accessed 22 May 2023.



For peer review only - http://bmjopen.bmj.com/site/about/guidelines.xhtml

37. Marteau TM, Bekker H. The development of a six-item short-form of the state scale of the Spielberger State—Trait Anxiety Inventory (STAI). Br J Clin Psychol. 1992;31(3):301-6.

38. Lerman C, Trock B, Rimer BK, Jepson C, Brody D, Boyce A. Psychological side effects of breast cancer screening. Health Psychol. 1991;10(4):259-67.

39. Weinstein ND. What does it mean to understand a risk? Evaluating risk comprehension. J Natl Cancer Inst Monographs. 1999;1999(25):15-20.

40. Ajzen I. Understanding attitudes and predicting social behavior. Englewood Cliffs, New Jersey: Prentice Hall; 1980.

41. French DP, Maissi E, Marteau TM. The psychological costs of inadequate cervical smear test results: three-month follow-up. Psychooncology. 2006;15(6):498-508.

42. Brehaut JC, O'Connor AM, Wood TJ, Hack TF, Siminoff L, Gordon E, et al. Validation of a decision regret scale. Med Decis Making. 2003;23(4):281-92.

43. Braun V, Clarke V. Using thematic analysis in psychology. Qual Res Psychol. 2006;3(2):77-101.

44. Braun V, Clarke V. Reflecting on reflexive thematic analysis. Qual Res Sport Exerc Health. 2019;11(4):589-97.

45. Pilgrim D. Some implications of critical realism for mental health research. Social Theory & Health. 2014;12(1):1-21.

46. NHS England. GP Patient Survey 2022: Technical Annex. 2022. https://www.gp-patient.co.uk/surveysandreports. Accessed 22 May 2023.

47. Malterud K, Siersma VD, Guassora AD. Sample size in qualitative interview studies: guided by information power. Qual Health Res. 2016;26(13):1753-60.

For peer review only



For peer review only - http://bmjopen.bmj.com/site/about/guidelines.xhtml

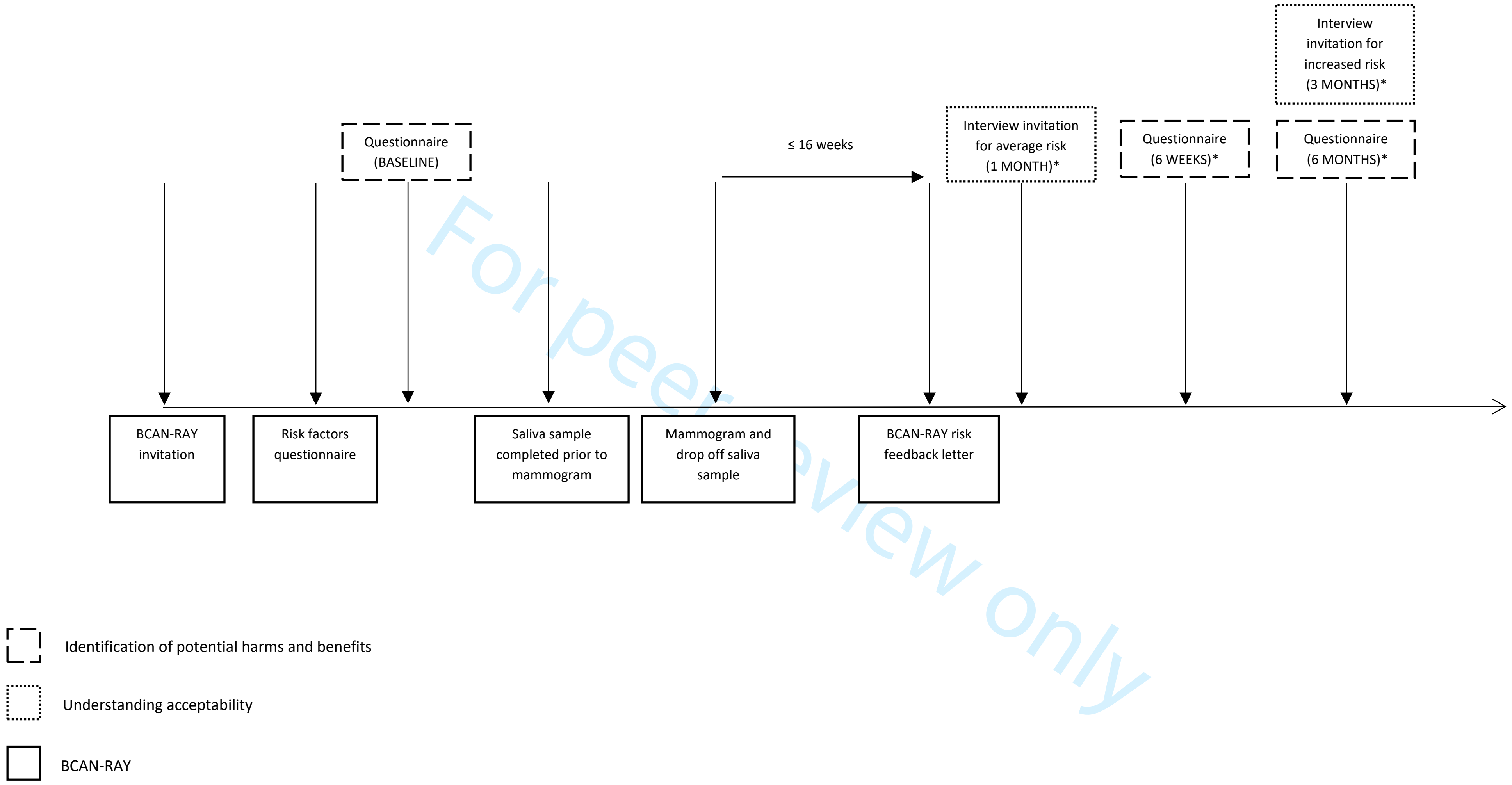


*Duration from risk feedback letter

**Supplementary file 1.** BCAN-RAY risk feedback letters (average, increased)

**INSERT LOGOS**

Nightingale Centre, Wythenshawe Hospital
Manchester University NHS Foundation Trust
Southmoor Road
Manchester
M23 9LT
**Tel: INSERT NUMBER**

**INSERT PARTICIPANT NAME**
**INSERT ADDRESS**
**INSERT ADDRESS**
**INSERT ADDRESS**

**INSERT POSTCODE**

Date: **INSERT DATE**

Dear **[INSERT NAME]**,

**RE: BCAN-RAY Study**
**NHS number: INSERT**

Thank you for taking part in the BCAN-RAY study. This is your first risk feedback letter. A second letter will follow when the study is complete for all women (probably in 2025). It is possible that the second letter may change your risk level.

We have calculated your risk of developing breast cancer in the next 10 years from the following information collected in this study:

- Breast cancer risk factors as assessed from the information you provided on the risk factor questionnaire
- Breast density (the amount of tissue in your breast that is not fat) as assessed from your mammogram
- DNA as assessed from your saliva (spit) sample

Your risk of developing breast cancer in the next 10 years was calculated to be:

**Average for the population – that is less than 3 in 100 chance of developing breast cancer in the next 10 years.**

More detailed information about your risk result is given in the enclosed document. This information is also available on the study web-based application, which can be accessed by scanning this QR code:

**INSERT QR CODE FOR WEB BASED APPLICATION**

We also confirm that no pathological variants (mutations) were identified in the 9 risk genes analysed in your saliva sample DNA.

For peer review only

**Further information and support resources**

There are things that all women can do to reduce their risk of breast cancer, such as maintaining a healthy weight through diet and exercise and limiting alcohol intake. More information on the ways to reduce your risk is provided in the accompanying leaflet. It is also important to regularly check your breasts and report anything new or unusual to a GP. A guide explaining how to check your breasts is enclosed.

Additionally, you may find the following sources of information and support useful if you have any breast health concerns.

**CoppaFeel!**

Website: https://coppafeel.org/

**Breast Cancer Now**

Website: https://breastcancernow.org/

They have a section where you can ask any questions you have relating to breast health:

https://forum.breastcancernow.org/t5/Ask-Our-Nurses/ct-p/Asknurses

They also offer a free, confidential helpline to answer questions about breast cancer or breast health – 0808 800 6000 (Text relay prefix – 18001)

**Should you have any questions about the study please get in touch with the study team on INSERT NUMBER**.

Yours sincerely,

**INSERT SIGNATURE**
**INSERT NAME**

For peer review only

INSERT LOGOS

Nightingale Centre, Wythenshawe Hospital
Manchester University NHS Foundation Trust
Southmoor Road
Manchester
M23 9LT
**Tel:** INSERT NUMBER

INSERT PARTICIPANT NAME
INSERT ADDRESS
INSERT ADDRESS
INSERT ADDRESS

INSERT POSTCODE

Date: INSERT DATE

Dear [INSERT NAME],

**RE: BCAN-RAY Study**
**NHS number:** INSERT

Thank you for taking part in the BCAN-RAY study. This is your first risk feedback letter.

A second letter will follow when the study is complete for all women (probably in 2025). It is possible that the second letter may change your risk level.

**Your result:**

You are at **increased** risk of breast cancer

This means that you are more likely to develop breast cancer than other women your age in the general population.

The details of your 10 year risk and lifetime risk of breast cancer compared to the general population are provided in the attached document and are also available on the study web-based application, which can be accessed by scanning this QR code:

INSERT QR CODE FOR WEB BASED APPLICATION

The factors that may have increased your personal risk were:

- Breast cancer risk factors as assessed from the information you provided on the risk factor questionnaire
- Breast density (the amount of tissue in your breast that is not fat) as assessed from your mammogram
- DNA as assessed from your saliva (spit) sample

At this level of risk you will be eligible to start breast screening earlier than the general population and will have access to breast cancer risk reducing approaches.

For peer review only

**Gene mutation search**

We did not identify a pathological variant (mutation) in any of the 9 risk genes tested.

OR

We have also identified a pathological variant (mutation) in one of the 9 risk genes tested. We would like to give you the opportunity to discuss the potential implications of this for yourself and your family in more detail and the planned risk review appointment (see below) will be with a geneticist (a doctor who specialises in gene mutations and what they mean for families).

**Risk review appointment**

We would like to offer you a face-to-face appointment at the Family History Risk and Prevention Clinic at The Nightingale Centre to discuss your risk result further. During this appointment, your breast cancer risk will be explained to you along with information about additional breast screening and when this can begin in addition to ways to reduce your risk.

This appointment is part of NHS care and not part of the study itself. As such, a referral into the clinic will be made by your GP and an appointment will be arranged. This should be within 8-12 weeks so if you have not received an appointment 8 weeks after receiving your risk result, please contact the Nightingale team on **INSERT NUMBER**.

**Further information and support resources**

There are things that all women can do to reduce their risk of breast cancer, such as maintaining a healthy weight through diet and exercise and limiting alcohol intake. More information on the ways to reduce your risk is provided in the accompanying leaflet. It is also important to regularly check your breasts and report anything new or unusual to a GP. A guide explaining how to check your breasts is enclosed.

Additionally, you may find the following sources of information and support useful if you have any breast health concerns.

**CoppaFeel!**

Website: https://coppafeel.org/

**Breast Cancer Now**

Website: https://breastcancernow.org/

They have a section where you can ask any questions you have relating to breast health:

https://forum.breastcancernow.org/t5/Ask-Our-Nurses/ct-p/Asknurses

They also offer a free, confidential helpline to answer questions about breast cancer or breast health – 0808 800 6000 (Text relay prefix – 18001)

**Should you have any questions about the study please get in touch with the study team on INSERT NUMBER.**

Yours sincerely,

**INSERT SIGNATURE**
**INSERT NAME**

**Supplementary file 2.** Detailed description of self-reported measures of potential harms and benefits of participation in breast cancer risk assessment

| Measures | Description |
| --- | --- |
| State anxiety (36) and cancer worry (37) | To determine whether increased distress is a harm of participating in breast cancer risk assessment, we will compare levels of general anxiety and breast cancer worry between average and increased risk women and across time to evaluate short as well as longer term effects. One might expect changes in distress, particularly amongst women being identified as increased risk, as the result may be unexpected because of a lack of family history of the disease. General state anxiety will be assessed using the six-item short-form of the state scale of the State Trait Anxiety Inventory (STAI) (36), with participants responding to six statements (e.g. "I feel tense") about how they currently feel by selecting one of the following response options "not at all", "somewhat", "moderately" and "very much".<br><br>Breast cancer worry will be assessed using the Lerman Cancer Worry Scale (37). The scale consists of six statements such as: "how often do you worry about developing breast cancer?". Participants will endorse one of the following response options for items 1-3 and 5: "never", "rarely", "sometimes", and "almost all the time". For items 4 and 6, participants select one option from "not at all", "a little", "somewhat", and "a lot". |

For peer review only

| | |
|---|---|
| | Both scales have previously been used in similar studies evaluating the psychological impact of receiving breast cancer risk estimates (26, 27). |
| Risk perception (38) | Perceived comparative risk of developing breast cancer will be assessed using a single item whereby women will be asked to rate their risk of developing breast cancer in the next 10 years, compared with other women of their age (38). Participants will select one of the following response options: “much higher”, “a bit higher”, “about the same”, “a bit lower”, and “much lower”. |
| Attitudes towards breast cancer risk assessment (39) | Attitudes towards breast cancer risk assessment will be assessed following a standard approach (39). Three items will be used to assess affective (feelings towards the behaviour) and instrumental (evaluation of the behaviour’s outcomes) attitudes. Women will be asked to indicate the extent to which they view risk assessment as good/beneficial/important, with response options including: “entirely good”, “mainly good”, “neither good nor bad”, “mainly bad”, and “entirely bad”. |
| Knowledge | No validated measure has been developed for the assessment of breast cancer risk assessment knowledge. Therefore, we decided to create a measure focusing on knowledge of the breast cancer risk assessment process to assess the potential benefit of increased knowledge and inform future implementation. The measure is informed by data on potential misunderstandings of the breast cancer risk assessment process |

For peer review only

| | |
|---|---|
| | identified from a content analysis of qualitative data collected in the context of optimising the delivery of breast cancer risk assessment in the BCAN-RAY study (28). The measure consists of three questions that map onto the potential misunderstandings identified, namely eligibility for risk assessment, the purpose of the mammogram and access to screening and preventive strategies. Subjective knowledge will be assessed with a single item that asks women to rate how informed they feel about their breast cancer risk, from "very well informed", "quite well informed", "quite uninformed", and "not very informed at all". |
| Satisfaction with risk feedback information (40) | Satisfaction with risk feedback information will be assessed using four items from a published scale (40) that has been used previously in breast cancer risk-stratification research (26, 27). Women will be asked how well informed they feel about their breast cancer risk, how satisfied they are with the amount of information given, how confusing they found it, and how clear they found the information. Participants will select one of the following response options for each item: "strongly agree", "agree", "agree somewhat", "undecided", "somewhat disagree", "disagree", and "strongly disagree. |
| Satisfaction with decision to participate in breast cancer risk assessment (41) | Participants' remorse or distress over their decision to take part in breast cancer risk assessment will be assessed using a single item adapted from the Decision Regret Scale (41): "The decision to participate in |

For peer review only

| | |
|---|---|
| | breast cancer risk assessment was a good decision for me". Response options will be "strongly agree", "agree", "neither agree nor disagree", "disagree", and "strongly disagree". |

For peer review only

**Supplementary file 3.** Participant questionnaires (baseline, 6 weeks post risk feedback and 6 months post risk feedback)

# **B**reast **CAN**cer – **R**isk Assessment in **Y**oung Women **(BCAN-RAY):**

## Acceptability survey (baseline)

**Please enter your unique identifier and date of birth. Your unique identifier can be found on your study invite letter.**

**Unique study identifier:**

**Date of birth:**

For peer review only

## SECTION A – YOUR MENTAL WELL-BEING

A number of statements which people have used to describe how they feel are given below. Please read each of the 6 statements and then circle the most appropriate number below the statement to indicate how you feel right now, at this moment. There are no right or wrong answers. Do not spend too much time on any one statement but give the answer which seems to describe your present feelings best.

**A1** **I feel calm**

| Not at all | Somewhat | Moderately | Very much |
|---|---|---|---|
| 1 | 2 | 3 | 4 |

**A2** **I am tense**

| Not at all | Somewhat | Moderately | Very much |
|---|---|---|---|
| 1 | 2 | 3 | 4 |

**A3** **I feel upset**

| Not at all | Somewhat | Moderately | Very much |
|---|---|---|---|
| 1 | 2 | 3 | 4 |

**A4** **I am relaxed**

| Not at all | Somewhat | Moderately | Very much |
|---|---|---|---|
| 1 | 2 | 3 | 4 |

**A5** **I feel content**

| Not at all | Somewhat | Moderately | Very much |
|---|---|---|---|
| 1 | 2 | 3 | 4 |

**A6** **I am worried**

| Not at all | Somewhat | Moderately | Very much |
|---|---|---|---|
| 1 | 2 | 3 | 4 |

For peer review only

**SECTION B – YOUR WORRIES ABOUT DEVELOPING BREAST CANCER**
Please read the statements below and circle the number below each statement that best indicates your current level of worry about getting breast cancer someday:

**B1** **How often have you thought about your chances of getting breast cancer?**

| Never | Rarely | Sometimes | Almost all the time |
|---|---|---|---|
| 1 | 2 | 3 | 4 |

**B2** **How often have these thoughts affected your mood?**

| Never | Rarely | Sometimes | Almost all the time |
|---|---|---|---|
| 1 | 2 | 3 | 4 |

**B3** **How often have these thoughts interfered with your ability to do daily activities?**

| Never | Rarely | Sometimes | Almost all the time |
|---|---|---|---|
| 1 | 2 | 3 | 4 |

**B4** **How concerned are you about the possibility of getting breast cancer one day?**

| Not at all | A little | Somewhat | A lot |
|---|---|---|---|
| 1 | 2 | 3 | 4 |

**B5** **How often do you worry about developing breast cancer?**

| Never | Rarely | Sometimes | Almost all the time |
|---|---|---|---|
| 1 | 2 | 3 | 4 |

**B6** **How much of a problem is this worry?**

| Not at all | A little | Somewhat | A lot |
|---|---|---|---|
| 1 | 2 | 3 | 4 |

For peer review only

**SECTION C – YOUR PERCEPTION OF BREAST CANCER RISK**
Please tick **ONE** of the statements below that best describes your breast cancer risk in relation to other women of a similar age:

**C1** **Compared to other women my age, I believe my risk of developing breast cancer in the next 10 years is…**

- ❑ Much higher
- ❑ A bit higher
- ❑ About the same
- ❑ A bit lower
- ❑ Much lower

**SECTION D – YOUR ATTITUDES TOWARD BREAST CANCER RISK ASSESSMENT**
Please read the statement and items below and circle the number that best indicates how you feel about participating in breast cancer risk assessment right now, at this moment:

**D1** **Taking part in breast cancer risk assessment will be…**

| | | | | |
|---|---|---|---|---|
| Entirely good | Mainly good | Neither good nor bad | Mainly bad | Entirely bad |
| 1 | 2 | 3 | 4 | 5 |
| Entirely beneficial | Mainly beneficial | Neither beneficial nor harmful | Mainly harmful | Entirely harmful |
| 1 | 2 | 3 | 4 | 5 |
| Entirely important | Mainly important | Neither important nor unimportant | Mainly unimportant | Entirely unimportant |
| 1 | 2 | 3 | 4 | 5 |

For peer review only

**SECTION E – INTEREST IN INTERVIEW**

We would like to hear more about your experience of participating in breast cancer risk assessment as part of the BCAN-RAY study. Please tick one box to indicate whether you are happy to be contacted about participating in an interview (over the phone or face-to-face).

**E1** **I am happy to be contacted about participating in an interview following receipt of my risk results**

YES ❑ NO ❑

***Thank you for completing this questionnaire.***

***Please return your completed questionnaire to the study team in the pre-paid envelope provided.***

**Sources of information and support**

You may find some of the following sources of information and support useful if you have any concerns about breast health.

**CoppaFeel!**

Website: https://coppafeel.org/

They have a section that provides guidance on checking your breasts: https://self-checkout.coppafeel.org/onboarding

**Breast Cancer Now**

Website: https://breastcancernow.org/

They have a section where you can ask any questions you have relating to breast health:

https://forum.breastcancernow.org/t5/Ask-Our-Nurses/ct-p/Asknurses

They also offer a free, confidential helpline to answer questions about breast cancer or breast health – 0808 800 6000 (Text relay prefix – 18001)

For peer review

# **B**reast **CAN**cer **– R**isk Assessment in **Y**oung Women **(BCAN-RAY):**

## Acceptability survey (6 weeks post risk feedback)

For peer review only

**Please enter your unique identifier and date of birth. Your unique identifier can be found on your study invite letter.**

**Unique study identifier:**

**Date of birth:**

**SECTION A – YOUR MENTAL WELL-BEING**

A number of statements which people have used to describe how they feel are given below. Please read each of the 6 statements and then circle the most appropriate number below the statement to indicate how you feel right now, at this moment. There are no right or wrong answers. Do not spend too much time on any one statement but give the answer which seems to describe your present feelings best.

**A1** **I feel calm**

| Not at all | Somewhat | Moderately | Very much |
|---|---|---|---|
| 1 | 2 | 3 | 4 |

**A2** **I am tense**

| Not at all | Somewhat | Moderately | Very much |
|---|---|---|---|
| 1 | 2 | 3 | 4 |

**A3** **I feel upset**

| Not at all | Somewhat | Moderately | Very much |
|---|---|---|---|
| 1 | 2 | 3 | 4 |

**A4** **I am relaxed**

| Not at all | Somewhat | Moderately | Very much |
|---|---|---|---|
| 1 | 2 | 3 | 4 |

**A5** **I feel content**

| Not at all | Somewhat | Moderately | Very much |
|---|---|---|---|
| 1 | 2 | 3 | 4 |

**A6** **I am worried**

| Not at all | Somewhat | Moderately | Very much |
|---|---|---|---|
| 1 | 2 | 3 | 4 |

For peer review only

For peer review only - http://bmjopen.bmj.com/site/about/guidelines.xhtml

**SECTION B – YOUR WORRIES ABOUT DEVELOPING BREAST CANCER**
Please read the statements below and circle the number below each statement that best indicates your current level of worry about getting breast cancer someday:

**B1** **How often have you thought about your chances of getting breast cancer?**

| Never | Rarely | Sometimes | Almost all the time |
|---|---|---|---|
| 1 | 2 | 3 | 4 |

**B2** **How often have these thoughts affected your mood?**

| Never | Rarely | Sometimes | Almost all the time |
|---|---|---|---|
| 1 | 2 | 3 | 4 |

**B3** **How often have these thoughts interfered with your ability to do daily activities?**

| Never | Rarely | Sometimes | Almost all the time |
|---|---|---|---|
| 1 | 2 | 3 | 4 |

**B4** **How concerned are you about the possibility of getting breast cancer one day?**

| Not at all | A little | Somewhat | A lot |
|---|---|---|---|
| 1 | 2 | 3 | 4 |

**B5** **How often do you worry about developing breast cancer?**

| Never | Rarely | Sometimes | Almost all the time |
|---|---|---|---|
| 1 | 2 | 3 | 4 |

**B6** **How much of a problem is this worry?**

| Not at all | A little | Somewhat | A lot |
|---|---|---|---|
| 1 | 2 | 3 | 4 |

For peer review only

**SECTION C – YOUR PERCEPTION OF BREAST CANCER RISK**
Please tick **ONE** of the statements below that best describes your breast cancer risk in relation to other women of a similar age:

**C1** **Compared to other women my age, I believe my risk of developing breast cancer in the next 10 years is…**

- ❑ Much higher
- ❑ A bit higher
- ❑ About the same
- ❑ A bit lower
- ❑ Much lower

**SECTION D – YOUR BREAST CANCER RISK KNOWLEDGE**
Please read the statement below and then circle the most appropriate number below the statement to indicate how informed you feel about your breast cancer risk at this moment:

**D1** **How informed do you feel about your breast cancer risk?**

| Very well informed | Quite well informed | Quite uninformed | Not very informed at all |
|---|---|---|---|
| 1 | 2 | 3 | 4 |

For peer review only

**SECTION E – YOUR KNOWLEDGE**

For each question please place **ONE** tick in the box that corresponds with your knowledge/understanding of breast cancer risk assessment being offered in the BCAN-RAY study.

**E1** **Who are the intended participants of breast cancer risk assessment in the BCAN-RAY study?**

- ❑ Women who have been told by a healthcare professional that they have a strong family history of breast cancer
- ❑ Women who have not been told by a healthcare professional that they have a strong family history of breast cancer

**E2** **What is the purpose of the low dose mammogram in the BCAN-RAY study?**

- ❑ To assess breast density (the amount of tissue in your breast that is not fat)
- ❑ To detect breast cancer

**E3** **Who will be given the opportunity to discuss additional breast screening and risk reducing measures with a clinician in the BCAN-RAY study?**

- ❑ Only women identified as being at increased risk of breast cancer
- ❑ All women who participate in the study

For peer review only

**SECTION F – YOUR PERCEPTIONS OF THE BREAST CANCER INFORMATION ENCLOSED WITH YOUR RISK FEEDBACK**

Thinking about the letter and leaflets you received when you were provided with your risk of developing breast cancer in the next 10 years, please read each statement and then circle the most appropriate number below the statement to indicate how you feel about the information *(please circle* ***only one number****).*

**F1** **I feel well informed about my breast cancer risk.**

| Strongly agree | Agree | Somewhat agree | Undecided | Disagree somewhat | Disagree | Strongly disagree |
|---|---|---|---|---|---|---|
| 1 | 2 | 3 | 4 | 5 | 6 | 7 |

**F2** **I feel satisfied with the amount of information I have been given.**

| Strongly agree | Agree | Somewhat agree | Undecided | Disagree somewhat | Disagree | Strongly disagree |
|---|---|---|---|---|---|---|
| 1 | 2 | 3 | 4 | 5 | 6 | 7 |

**F3** **I am confused by the information I have been given.**

| Strongly agree | Agree | Somewhat agree | Undecided | Disagree somewhat | Disagree | Strongly disagree |
|---|---|---|---|---|---|---|
| 1 | 2 | 3 | 4 | 5 | 6 | 7 |

**F4** **The information was clear.**

| Strongly agree | Agree | Somewhat agree | Undecided | Disagree somewhat | Disagree | Strongly disagree |
|---|---|---|---|---|---|---|
| 1 | 2 | 3 | 4 | 5 | 6 | 7 |

***Thank you for completing this questionnaire.***

***Please return your completed questionnaire to the study team in the pre-paid envelope provided.***

For peer review only

## Sources of information and support

You may find some of the following sources of information and support useful if you have any concerns about breast health.

**CoppaFeel!**

Website: https://coppafeel.org/

They have a section that provides guidance on checking your breasts: https://self-checkout.coppafeel.org/onboarding

**Breast Cancer Now**

Website: https://breastcancernow.org/

They have a section where you can ask any questions you have relating to breast health:

https://forum.breastcancernow.org/t5/Ask-Our-Nurses/ct-p/Asknurses

They also offer a free, confidential helpline to answer questions about breast cancer or breast health – 0808 800 6000 (Text relay prefix – 18001)

For peer review only

# **B**reast **CAN**cer **– R**isk Assessment in **Y**oung Women **(BCAN-RAY):** Acceptability survey (6 months post risk feedback)

**Please enter your unique identifier and date of birth. Your unique identifier can be found on your study invite letter.**

**Unique study identifier:**

**Date of birth:**

For peer review only

**SECTION A – YOUR MENTAL WELL-BEING**
A number of statements which people have used to describe how they feel are given below. Please read each of the 6 statements and then circle the most appropriate number below the statement to indicate how you feel right now, at this moment. There are no right or wrong answers. Do not spend too much time on any one statement but give the answer which seems to describe your present feelings best.

**A1** **I feel calm**

| Not at all | Somewhat | Moderately | Very much |
|---|---|---|---|
| 1 | 2 | 3 | 4 |

**A2** **I am tense**

| Not at all | Somewhat | Moderately | Very much |
|---|---|---|---|
| 1 | 2 | 3 | 4 |

**A3** **I feel upset**

| Not at all | Somewhat | Moderately | Very much |
|---|---|---|---|
| 1 | 2 | 3 | 4 |

**A4** **I am relaxed**

| Not at all | Somewhat | Moderately | Very much |
|---|---|---|---|
| 1 | 2 | 3 | 4 |

**A5** **I feel content**

| Not at all | Somewhat | Moderately | Very much |
|---|---|---|---|
| 1 | 2 | 3 | 4 |

**A6** **I am worried**

| Not at all | Somewhat | Moderately | Very much |
|---|---|---|---|
| 1 | 2 | 3 | 4 |

For peer review only

**SECTION B – YOUR WORRIES ABOUT DEVELOPING BREAST CANCER**
Please read the statements below and circle the number below each statement that best indicates your current level of worry about getting breast cancer someday:

**B1** **How often have you thought about your chances of getting breast cancer?**

| Never | Rarely | Sometimes | Almost all the time |
|---|---|---|---|
| 1 | 2 | 3 | 4 |

**B2** **How often have these thoughts affected your mood?**

| Never | Rarely | Sometimes | Almost all the time |
|---|---|---|---|
| 1 | 2 | 3 | 4 |

**B3** **How often have these thoughts interfered with your ability to do daily activities?**

| Never | Rarely | Sometimes | Almost all the time |
|---|---|---|---|
| 1 | 2 | 3 | 4 |

**B4** **How concerned are you about the possibility of getting breast cancer one day?**

| Not at all | A little | Somewhat | A lot |
|---|---|---|---|
| 1 | 2 | 3 | 4 |

**B5** **How often do you worry about developing breast cancer?**

| Never | Rarely | Sometimes | Almost all the time |
|---|---|---|---|
| 1 | 2 | 3 | 4 |

**B6** **How much of a problem is this worry?**

| Not at all | A little | Somewhat | A lot |
|---|---|---|---|
| 1 | 2 | 3 | 4 |

For peer review only

**SECTION C – YOUR PERCEPTION OF BREAST CANCER RISK**
Please tick **ONE** of the statements below that best describes your breast cancer risk in relation to other women of a similar age:

**C1** **Compared to other women my age, I believe my risk of developing breast cancer in the next 10 years is…**

- ❑ Much higher
- ❑ A bit higher
- ❑ About the same
- ❑ A bit lower
- ❑ Much lower

**SECTION D – YOUR ATTITUDES TOWARD BREAST CANCER RISK ASSESSMENT**
Please read the statement and items below and circle the number that best indicates how you feel about participating in breast cancer risk assessment right now, at this moment:

**D1** **Taking part in breast cancer risk assessment was…**

| | | | | |
|---|---|---|---|---|
| Entirely good | Mainly good | Neither good nor bad | Mainly bad | Entirely bad |
| 1 | 2 | 3 | 4 | 5 |
| Entirely beneficial | Mainly beneficial | Neither beneficial nor harmful | Mainly harmful | Entirely harmful |
| 1 | 2 | 3 | 4 | 5 |
| Entirely important | Mainly important | Neither important nor unimportant | Mainly unimportant | Entirely unimportant |
| 1 | 2 | 3 | 4 | 5 |

For peer review only

**SECTION E – YOUR SATISFACTION WITH DECISION TO PARTICIPATE IN BREAST CANCER RISK ASSESSMENT**

Please read the statement below and then circle the most appropriate number below the statement to indicate how satisfied you are with your decision to participate in breast cancer risk assessment.

**E1** **The decision to participate in breast cancer risk assessment was a good decision for me**

| Strongly agree | Agree | Neither agree nor disagree | Disagree | Strongly disagree |
|---|---|---|---|---|
| 1 | 2 | 3 | 4 | 5 |

For peer review

***Thank you for completing this questionnaire.***

***Please return your completed questionnaire to the study team in the pre-paid envelope provided.***

**Sources of information and support**

You may find some of the following sources of information and support useful if you have any concerns about breast health.

**CoppaFeel!**

Website: https://coppafeel.org/

They have a section that provides guidance on checking your breasts: https://self-checkout.coppafeel.org/onboarding

**Breast Cancer Now**

Website: https://breastcancernow.org/

They have a section where you can ask any questions you have relating to breast health:

https://forum.breastcancernow.org/t5/Ask-Our-Nurses/ct-p/Asknurses

They also offer a free, confidential helpline to answer questions about breast cancer or breast health – 0808 800 6000 (Text relay prefix – 18001)

**Supplementary file 4.** Interview topic guide

**Opening questions**

As you know, we are interested in what women think about the offer of finding out their breast cancer risk as part of the BCAN-RAY study. To start, can you tell me anything about whether breast cancer risk is something you have thought about before being invited to join the BCAN-RAY study?

I understand you were invited to have your breast cancer risk assessed; can we go back to that point and tell me what that was like? What did you think at that point?

How did you make the decision to take part in breast cancer risk assessment?
Prompts:

- Were there are aspects of the BCAN-RAY study that made you question whether to take part (any concerns)?
- Can you tell me anything about why you wanted to know your risk? Anything personal to you?
- How did you receive the invite (as a letter from GP practice if no recall)? What do you think about receiving it that way? How do you think that influenced your decision to have your breast cancer risk assessed?
- (if not already come up) When you were deciding, did you discuss it with anyone (friend / family / study team / GP)?
- Did you feel you had all the information you needed to make a decision about whether to take part? If not, what would have been helpful to know?

**Questions relating to risk assessment process**

Can you tell me what you had to do once you joined the study? Could you tell me about what happened when you had your breast cancer risk assessed?
Probes: What was it like / can you tell me anything about it
Prompts:

- Completing the risk factors questionnaire e.g. how easy was it to access, can you remember what it was asking you to do, were any questions unclear, ability to answer the questions more generally, did you find any questions uncomfortable to answer, did you get any support to help with this part of the study
- What happened once you completed the questionnaire? What was that time-period like?
- Attending the appointment at the hospital (spit sample, mammogram) e.g. what did you think about how the appointment was arranged
- Waiting for the risk feedback results (up to 16 weeks turnaround) e.g. how were you feeling during this time, what did you think about the length of time you had to wait, did you look for any information related to breast cancer during this time
- Receiving the risk feedback (a letter if no recall)
- Contents/wording of the letter (thoughts, feelings and understanding) e.g. did the feedback you received match your expectations in terms of what you thought you would be told
- Logging back into the app to view detailed risk feedback (if not, why not)

For peer review only

- Personal meaning of risk category received e.g. what do you remember about your risk result, how would you describe the risk, how do you feel about the factors that contributed to your risk (increased risk), how did it make you feel, was it something you expected, how do you feel about your risk today/now
- Discussing risk feedback with others (friends / family / healthcare professionals)
  - Did you talk about your risk feedback with anyone in the study team / outside the study team? If yes/no, why? What did you discuss?
  - What did you think of the support provided at this point?
- (increased risk) Experience of risk consultation
  - What did you think about the option to receive an appointment to discuss your risk if it was increased?

After you received your risk feedback, did you do anything differently that you thought might reduce your breast cancer risk?
Prompts:

- (all) Health behaviours
- (increased risk) Recommendation to contact medical doctors to discuss risk reducing medication / additional screening
- (increased risk) Deciding whether to have risk reducing medication
- (increased risk) Deciding whether to have additional screening

Looking back, was there anything that caused any concerns during the risk assessment process? Is there anything you would have preferred to happen in a different way?

Looking back, how do you feel about having made the decision to take part in breast cancer risk assessment?
Prompts:

- Did you understand what was involved when you made the decision to participate?
  Probe: did you have sufficient information?

The way breast cancer risk is calculated changes over time as we learn more about new risk factors. As we are trying to find out whether using a low dose mammogram helps to identify younger women at risk of developing breast cancer, towards the end of the study you will receive updated risk feedback. At this point, your risk might change. What are your thoughts about this? Why?

We are trying to figure out whether introducing a breast cancer risk assessment service for women aged 30 to 39 years is a good or bad idea. What are your thoughts about this? Why? Would you recommend a breast cancer risk assessment service to friends and family members of a similar age?

**Finishing comments**

Thanks for your time today. We do really appreciate it.

- Is there anything else you want to add?

For peer review only

- Is there anything you thought you would talk about today which you haven't had a chance to say and want to mention?
- Do you have any questions for me?

Thanks again. The interview will be typed up by a partner transcription company we use. When this is done, we will remove anything you have said that could identify you such as names or places and you will be given a fake name. If you have any questions feel free to contact the research team at any time *[point out contact details]*.

For peer review only
